# Genetic Insights Into Perinatal Outcomes of Maternal Antihypertensive Therapy During Pregnancy

**DOI:** 10.1001/jamanetworkopen.2024.26234

**Published:** 2024-08-27

**Authors:** Ciarrah-Jane S. Barry, Venexia M. Walker, Christy Burden, Alexandra Havdahl, Neil M. Davies

**Affiliations:** 1 Medical Research Council Integrative Epidemiology Unit, University of Bristol, Bristol, United Kingdom; 2 Population Health Sciences, Bristol Medical School, University of Bristol, Bristol, United Kingdom; 3 Department of Surgery, University of Pennsylvania Perelman School of Medicine, Philadelphia; 4 Translational Health Sciences, Bristol Medical School, University of Bristol, Bristol, United Kingdom; 5 Nic Waals Institute, Lovisenberg Diaconal Hospital, Oslo, Norway; 6 Center for Genetic Epidemiology and Mental Health, Norwegian Institute of Public Health, Oslo, Norway; 7 PROMENTA, Department of Psychology, University of Oslo, Oslo, Norway; 8 K.G. Jebsen Center for Genetic Epidemiology, Department of Public Health and Nursing, Norwegian University of Science and Technology, Trondheim, Norway; 9 Division of Psychiatry, University College London, London, United Kingdom; 10 Department of Statistical Science, University College London, London, United Kingdom

## Abstract

**Importance:**

Limited information exists regarding the impact of pharmacotherapy in pregnancy due to ethical concerns of unintended fetal harm. Yet, maternal prescriptive drug use for chronic conditions such as hypertension is common.

**Objective:**

To investigate potential causal relationships between perturbing maternal genetic variants influencing antihypertensive drug targets and perinatal outcomes among offspring using mendelian randomization (MR).

**Design, Setting, and Participants:**

This 2-sample MR study used individual-level single-nucleotide variation (SNV) outcome data from mother-father-offspring trios with complete genetic and phenotypic information from the Norwegian Mother, Father and Child Cohort Study (MoBa) and summary-level SNV exposure data from UK Biobank participants sourced from the Integrative Epidemiology Unit OpenGWAS project. Pregnant individuals were recruited across Norway during their routine ultrasonography examination at 18 weeks’ gestation between June 1999 and December 2008, and mothers, fathers, and offspring were followed up after birth. Novel genetic instruments for maternal antihypertensive drug targets that act via systolic blood pressure (SBP) were derived from individual-level data analyzed in January 2018. Two-sample multivariable MR analysis of these maternal drug targets and offspring outcomes were performed between January 2023 and April 2024.

**Exposures:**

Maternal genetic variants associated with drug targets for treatments of hypertension, as specified in the National Health Service dictionary of medicines and devices.

**Main Outcomes and Measures:**

Offspring outcomes were Apgar score at 1 minute and 5 minutes, offspring developmental score at 6 months, birth length, birth weight *z *score, gestational age, head circumference, and congenital malformation. Maternal hypertensive disorders of pregnancy were a positive control.

**Results:**

The MoBa sample contained 29 849 family trios, with a mean (SD) maternal age of 30.2 (18.6) years and a mean (SD) paternal age of 32.8 (13.1) years; 51.1% of offspring were male. Seven independent SNVs were identified as influencing maternal SBP via the antihypertensive drug target instruments. For higher levels of maternal SBP acting through the *CACNB2* calcium channel blocker target, the estimated change in gestational age was 3.99 days (95% CI, 0.02-7.96 days) per 10-mm Hg decrease in SBP. There was no evidence of differential risk for measured perinatal outcomes from maternal SBP acting through drug targets for multiple hypertensive subclasses, such as between the *ADRB1* β-adrenoceptor–blocking target and risk of congenital malformation (estimated odds ratio, 0.28 [95% CI, 0.02-4.71] per 10-mm Hg decrease in SBP). Maternal and paternal SBP acting through the *EDNRA* vasodilator antihypertensive target did not have a potential causal effect on birth weight *z* score, with respective β estimates of 0.71 (95% CI, −0.09 to 1.51) and 0.72 (95% CI, −0.08 to 1.53) per 10-mm Hg decrease in SBP.

**Conclusions and Relevance:**

The findings provided little evidence to indicate that perturbation of maternal genetic variants for SBP that influence antihypertensive drug targets had potential causal relationships with measures of perinatal development and health within this study. These findings may be triangulated with existing literature to guide physicians and mothers in decisions about antihypertensive use during pregnancy.

## Key Points

**Question:**

Is there a potential causal relationship between maternal antihypertensive drug exposure during pregnancy and offspring outcomes?

**Findings:**

In this mendelian randomization study including 29 849 mother-father-offspring trios, maternal genetic variants for systolic blood pressure acting through drug targets for treatments of hypertension provided little evidence for potential causal relationships between multiple antihypertensive drug subclass targets and differential risk of measured perinatal outcomes. Paternal and maternal effect estimates were similar.

**Meaning:**

This study found little evidence of harm to offspring from the studied antihypertensive subclasses and may establish a framework to assess the risks of the impact of maternal drug target perturbation during pregnancy using family-based genetic data.

## Teaser

This mendelian randomization study uses mendelian randomization to investigate potential causal relationships between perturbing maternal genetic variants influencing antihypertensive drug targets and perinatal outcomes among offspring.

## Introduction

Preexisting chronic and acute conditions may require therapeutic management during pregnancy to avoid adverse maternal outcomes. Approximately 8% to 10% of pregnancies globally are affected by hypertension or high blood pressure, including chronic hypertension (preexisting, typically essential), gestational hypertension (new after 20 weeks’ gestation), and preeclampsia (hypertension with additional features of multiorgan involvement).^1,2,3^ When left untreated, these are well-established risk factors for numerous serious adverse maternal and early infant outcomes.^1,4^ These include preeclampsia, maternal death, preterm birth, intrauterine growth restriction, low birth weight, and neuropsychiatric disorders.^1,4,5^ Yet, treatments for these conditions have been associated with possible additional risks for the developing fetus (eTable 1 in Supplement 1).

Profound physiologic changes occur during pregnancy, such as increased body weight, kidney blood flow, and cardiac output.^6,7,8^ These changes have been found or theorized to impact the pharmacokinetics of many drugs, affecting distribution, absorption, and metabolism.^7,8,9^ Yet, clinical trials typically exclude pregnant individuals. Recruiting pregnant individuals to randomized clinical trials (RCTs) is challenging for ethical and practical reasons. Additionally, experiments using pregnant animals are of limited relevance as there may be species-specific effects, whereby a drug is harmful in some animal species but not in a human or vice versa.^10^ As a result, there is relatively little evidence about the effects of drugs in pregnancy both on mothers and on their offspring. Observational studies on pregnant individuals have found evidence of possible impaired perinatal development due to drug exposure (teratogenic effects).^11,12,13^ However, the data are limited and often conflicting. Clinical guidance is developed using limited available pharmacologic evidence, typically with a tendency toward conservative behavior. Thus, fear of unintentional fetal harm potentially puts mothers and their offspring at risk through medication avoidance or nonadherence.^14^ In the UK, the most widely used and recommended antihypertensive drug in pregnancy is labetalol (β-adrenoreceptor blocker), but its use has been associated with fetal growth restriction and perinatal hypoglycemia.^13,15,16^ Nifedipine (calcium channel blocker) is the second most used antihypertensive drug in pregnancy, followed by methyldopa, neither of which is routinely recommended outside pregnancy. Typically, evidence has shown low adherence to antihypertensive medication in pregnancy, which can put the mother and fetus at risk.^17,18^

Genetics can provide a complementary source of evidence about the effects of drugs in utero. Mendelian randomization (MR) is an instrumental variables analysis in which genetic variants associated with the exposure of interest are used to assess the potential causal relationships between an exposure and an outcome.^19^ A genetic variant fulfills the criteria of a valid instrument if it (1) is reliably associated with the exposure of interest (relevance), (2) has no uncontrolled common cause with the outcome relationship (independence), and (3) is only associated with the outcome via its effect on the exposure of interest (exclusion restriction).

Drug target MR, which uses genetic variants within a gene to proxy a protein drug target, can potentially be used to assess drug safety during pregnancy without exposing the fetus to additional risks.^20^ In this study, we were interested in the intrauterine effects of drugs taken by the mother during pregnancy; hence, we used an intergenerational, within-family MR design in a large dataset of genotyped parent-offspring trios. If the MR assumptions held, the maternal genotype would be a proxy for prescription drug exposure in utero.

Genetic variants are randomly transmitted from parents to offspring at conception and thus cannot be affected by external factors after conception. However, exposure-related segregation distortion may exist, meaning that environmental factors or downstream expression of variants may influence the probability of a genetic variant being transmitted from the parent via a successful pregnancy.^19,21,22^ Without related segregation distortion, this random allocation of genetic exposure is analogous to the randomization of treatment within an RCT. As a result, conditional on parental genotype and in the absence of selection bias, offspring genotypes will be independent of the preconception environment.

**Figure 1.**
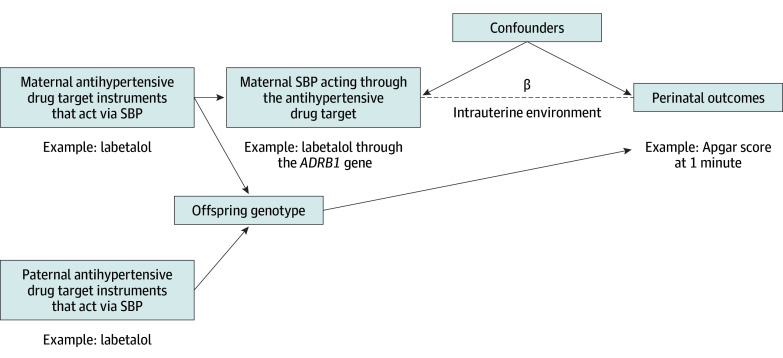
Directed Acyclic Graph Demonstrating the Intergenerational Within-Family Pharmacologic Mendelian Randomization Framework Arrows indicate offspring outcomes mediated via phenotypic expression in the offspring or the genetic inheritance of the offspring from the mother. β Represents the estimate of interest. Maternal exposure to a drug target of interest, such as a β-adrenoreceptor blocker, is instrumented by the maternal genotype in a gene (eg, the single-nucleotide variations on the *ADRB1* gene targeted by the drug substance). The offspring genotype may directly influence the offspring outcome; thus, an estimate that controls for offspring genotype would be unbiased by direct genetic inheritance as it would close the potential direct causal pathway and ensure the independence assumption holds. SBP indicates systolic blood pressure.

It is possible to instrument drug exposure using genetic variants within genes that relate to the activity of or encode the protein target of the drug.^20^ Intergenerational, within-family MR uses genetic variants in 1 generation (eg, the mother’s genotype) as an instrument for assessing maternal exposure to estimate the effects on the offspring.^23,24^ Thus, maternal genetic variants related to the activity or expression of a drug target or biomarker may be used as a proxy for drug exposure effects on infant outcomes to examine evidence of potential teratogenic or beneficial effects (Figure 1).^20,25^

Mendelian randomization has been implemented in the literature to identify opportunities for drug repurposing, drug targets, and estimating adverse drug effects.^20,26,27,28,29,30,31^ However, few studies have used MR to estimate the intrauterine effects of drugs. Evidence from MR should be triangulated with findings from other study designs to guide clinical decision-making, develop or repurpose drugs, or establish drug safety profiles. In this study, we developed and implemented a novel instrument derivation method to investigate whether potential causal relationships of intrauterine antihypertensive prescriptive drug exposure with offspring outcomes may be estimated.

## Methods

### Study Design

In this 2-sample MR study, conducted between January 2023 and April 2024, we assessed the potential causal relationship of genetically proxied intrauterine drug exposure with outcomes among offspring. Details on how this study fits the Strengthening the Reporting of Observational Studies in Epidemiology Using Mendelian Randomization (STROBE-MR) guidelines are provided in eAppendix 1 in Supplement 1. Summary-level single-nucleotide variation (SNV)–exposure associations were extracted from a systolic blood pressure GWAS conducted in UK Biobank and accessed through the Integrative Epidemiology Unit (IEU) OpenGWAS platform.^32^ These estimates were calculated within a sample of 436 419 male and female European participants and were not adjusted for measures of body weight or medication use.^33,34^ The current study was approved by Norway’s Regional Committees for Medical and Health Research Ethics. Written informed consent was provided by all participants at recruitment.

We derived the SNV-outcome associations using individual-level data from the Norwegian Mother, Father and Child Cohort Study (MoBa), a prospective, population-based pregnancy cohort study conducted by the Norwegian Institute of Public Health. Pregnant individuals across Norway were recruited at approximately 18 weeks’ gestation between June 1999 and December 2008.^35,36^ The individuals consented to participation in 41% of the invited pregnancies. The MoBa cohort includes 114 761 children, 95 248 mothers, and 74 626 fathers. The establishment of MoBa and initial data collection were based on a license from the Norwegian Data Protection Authority and approval from the Regional Committees for Medical and Health Research Ethics. The Norwegian Health Registry Act currently regulates the MoBa cohort.

Data collection occurred at multiple time points during the pregnancy in the form of self-reported questionnaires that were continued after birth. English translations of the questionnaires are available online.^37^ The MoBa data in this study used version 12 of the quality-assured data, released in January 2019. Additional information regarding the child’s birth record is available from linkage to the Medical Birth Registry of Norway (MBRN), the national compulsory registry containing information about all births in Norway since 1967.^35,36,38^ Blood samples were obtained from both parents during pregnancy and from mothers and neonates (umbilical cord) at birth.^39^ Genetic data were available for 44 017 mother-father-child trios, with details on genotyping, imputation, and quality control available elsewhere.^40^

The specific data of interest to this study were from birth information from the MBRN, the questionnaire 6 months after birth, and genotyped data from mother, father, and offspring trios. We restricted the sample to parent-offspring trios with complete genetic and outcome covariate data (eFigure 1 in Supplement 1).

### Exposures

Using the National Health Service (NHS) dictionary of medicines and devices search on OpenPrescribing, hypertension-related virtual medicinal products (VMPs) were determined via British National Formulary (BNF) codes (eAppendix 2 in Supplement 1).^41,42^ The BNF codes and corresponding drug subclasses determined to be relevant to hypertension are listed in Table 1. Drug substances known to be prescribed for hypertension without an allocated BNF code in the OpenPrescribing dictionary of medicines and devices were subsequently manually added (meprobamate, potassium chloride, tadalafil, and tamsulosin).

**Table 1.** British National Formulary Codes Used to Search the National Health Service Dictionary of Medicines and Devices to Determine Drug Substances Relevant to the Study

Code	Drug subclass
020201	Thiazides and related diuretics
020202	Loop diuretics
020203	Potassium-sparing diuretics and aldosterone antagonists
02040	β-Adrenoceptor–blocking drugs
02050	Vasodilator antihypertensive drugs, centrally acting antihypertensive drugs, adrenergic neuron–blocking drugs, α-adrenoceptor–blocking drugs, renin-angiotensin system drugs, angiotensin-converting enzyme inhibitors, angiotensin II receptor antagonists, renin inhibitors, ganglion-blocking drugs, other adrenergic neuron–blocking drugs
020602	Calcium channel blockers
040102	Anxiolytics

### Instrument Selection

The active drug substance was identified in each exposure VMP (eTable 2 in Supplement 1). For each drug substance, the corresponding pharmacologically active gene was identified in DrugBank.^43^ Using GENCODE, each gene was mapped to its corresponding genome location (chromosome:base pair range) as indicated by release 43 of the Genome Reference Consortium Human Build 37 assembly.^44^ All SNVs within these genomic regions were extracted from the MoBa trio dataset for mothers, fathers, and offspring.

### Outcomes

A binary measure for hypertensive disorders of pregnancy was derived as a positive control and was set to “yes” if there was evidence within the following MBRN variables: hypertension in pregnancy, eclampsia, preeclampsia, early preeclampsia, and hemolysis, elevated liver enzymes, and low platelets syndrome.^45^ The Ages and Stages Questionnaire items in the 6-month questionnaire were used to calculate an offspring developmental score (details in eAppendix 3 in Supplement 1). Thus, the outcomes of interest were hypertensive disorders of pregnancy, perinatal birth weight for gestational age *z *score (hereafter, *birth weight z score*), gestational age (days), length at birth (cm), head circumference (cm), Apgar score at 1 minute, Apgar score at 5 minutes, developmental score at 6 months (score range, 0-10, with higher scores indicating better development), and congenital malformation (eTable 3 in Supplement 1).

Each SNV-outcome association was estimated using linear or logistic regression for continuous or binary measures. To ensure the independence assumption was met, paternal and offspring genotypes were included in the regression alongside the maternal genotype. Within each model, we also controlled for offspring sex, parental age, batch effects, and the top 20 principal components.

### Exposure Data

Summary-level data from a genome-wide association study (GWAS) of systolic blood pressure (SBP) within UK Biobank were used as the exposure dataset (details in eAppendix 4 in Supplement 1). The sample contained 436 419 female and male participants of European ancestry. We selected the variants that were common to both MoBa and UK Biobank. We then identified the subset of SNVs that were associated with SBP (*P* < 5 × 10^−8^) and clumped them with a linkage disequilibrium threshold of *r*^2^ < 0.01.

### Statistical Analysis

We used a 2-sample multivariable MR analysis to estimate the potential causal relationship of the derived maternal drug proxies with early infant outcomes. We used R, version 4.3.0 (R Project for Statistical Computing) to analyze the data via the TwoSampleMR package.^46,47^ The datasets were harmonized, and palindromic SNVs that were not inferable from their allele frequency were discarded (SNVs having a minor allele frequency >0.42).

In MR analyses, our hypothesis tests were 2-sided. We evaluated estimates by considering precision, effect estimate direction, and sample size to prevent the misinterpretation of statistical significance based solely on *P* values, as indicative findings may warrant further study.^49^ We estimated the potential causal relationship of a 10-mm Hg decrease in SBP with each outcome using the Wald estimator for drug classes with a single SNV. For drug subclasses with multiple SNVs, we first checked the mechanism of action of all genes targeted by each SNV for the drug subclass. If the mechanisms of action were identical, we used the inverse variance weighted (IVW) estimator for each outcome. If the mechanisms of action were conflicting, SNVs were subdivided into their mechanistic groupings. Coefficient estimates for binary measures were exponentiated and reported as odds ratios (ORs) per 10-mm Hg decrease in SBP, as this is comparable to the effect of using an antihypertensive drug.^48^

As a sensitivity analysis, we used the paternal genotype as a negative control to determine whether the SNVs were acting on the offspring via the maternal genotype.^50^ We estimated the potential causal relationship of paternal genotype with the offspring outcome while controlling for the maternal and offspring genotypes. If the potential effects of variants on the offspring outcomes were due to the intrauterine environment, we would expect the maternal but not the paternal variants to be associated with the outcomes.

Additionally, we tested the relevance assumption by calculating the individual and mean *F* statistics of the instrument-exposure association. An *F* statistic greater than 10 was indicative that the model was unlikely to have substantial weak instrument bias.^51^

## Results

### Instrument Derivation

There were 29 849 complete MoBa trios in our study, with mean (SD) maternal age of 30.2 (18.6) years and paternal age of 32.8 (13.1) years. Of the offspring, 48.9% were female and 51.1% were male. A total of 918 mothers in MoBa overall and 228 of the current study’s cohort of trios (0.8%) reported antihypertensive drug use at any point during the pregnancy. Summary details of outcome measures are available in eTable 3 in Supplement 1, exclusion criteria are shown in eFigure 1 in Supplement 1, and a description of the cohort characteristics is available in the cohort profile.^35,36^

The overall instrument derivation process is displayed in eFigure 2 in Supplement 1. Details of the demographic characteristics of the sample are available in eTable 3 in Supplement 1. In this study, instruments were selected from 23 022 SNVs in 73 uniquely identified genomic regions. The unrestricted SNV-exposure SBP GWAS contained 9 837 128 SNVs. Of those, 21 535 (2.2%) were available in both the SNV-exposure and the SNV-outcome datasets. After applying the GWAS *P* value threshold (*P* < 5 × 10^−8^), 292 SNVs (1.4%) remained. These SNVs were then clumped to ensure the estimation of statistically independent signals. After all exclusions, we obtained 7 SNVs as instruments (Table 2).

**Table 2.** Single-Nucleotide Variation Identification Numbers, Drug Substances, and Corresponding Genes Used in the Mendelian Randomization Analysis

rsID	Drug subclass	BNF code	Gene
rs10764331, rs12258967, rs3821843	Calcium channel blockers	206020	*CACNB2*
rs1262894	Potassium-sparing diuretics and aldosterone antagonists	202030	*SCNN1D*
rs13143677	Vasodilator antihypertensive drugs targeting *EDNRA*	205010	*EDNRA*
rs1557765	Vasodilator antihypertensive drugs targeting *KNCJ11*	205010	*KCNJ11*
rs1801253	β-Adrenoceptor–blocking drugs	2040	*ADRB1*

Abbreviations: BNF, British National Formulary; rsID, single-nucleotide variation identification number.

### Intrauterine Effects

**Figure 2.**
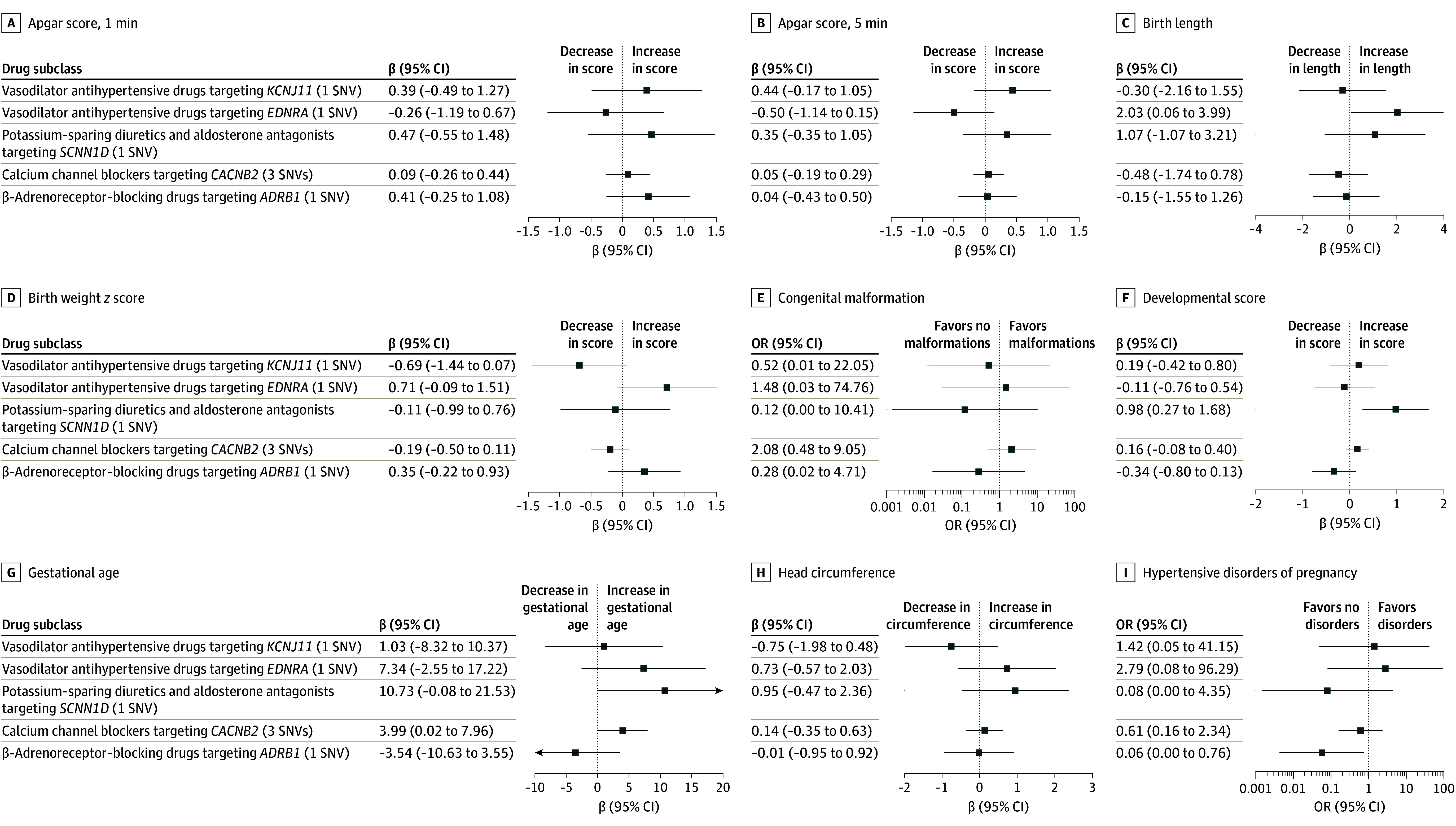
Forest Plots Demonstrating the Estimated Causal Effect of the Maternal Genetic Drug Targets With Offspring Outcomes per 10-mm Hg Decrease in Systolic Blood Pressure Results are shown for the inverse variance–weighted estimate where multiple single-nucleotide variations (SNVs) were available and for the Wald ratio otherwise. Odds ratios (ORs) were estimated for the outcome of hypertensive disorders of pregnancy. All other estimates are mean differences.

Figure 2 and eTable 4 in Supplement 1 display results for the potential effects of maternal SBP acting through antihypertensive drug targets on offspring outcomes. Across all available drug subclasses, there was some evidence from our analyses that maternal SBP acting through targets of antihypertensive drugs potentially affected early infant outcomes.

#### Birth Length and Weight

Estimates for the change in birth length (Figure 2C) varied by target. Estimates ranged from −0.48 cm (95% CI, −1.74 to 0.78 cm) per 10-mm Hg decrease in maternal SBP acting through *CACNB2*, a target of calcium channel blockers, to 2.03 cm (95% CI, 0.06-3.99 cm) per 10-mm Hg decrease in maternal SBP acting through *EDNRA*, a target of vasodilator antihypertensives. Similarly, estimates for the change in birth weight *z* score (Figure 2D) ranged from −0.69 (95% CI, −1.44 to 0.07) per 10-mm Hg decrease in maternal SBP acting through *KCNJ11*, a target of vasodilator antihypertensives, to 0.71 (95% CI, −0.09 to 1.51) per 10-mm Hg decrease in maternal SBP acting through *EDNRA*, another target of vasodilator antihypertensives.

#### Congenital Malformation

Estimates for congenital malformation are shown in Figure 2E. The ORs ranged from 0.12 (95% CI, <0.01 to 10.41) per 10-mm Hg decrease in maternal SBP acting through *SCNN1D*, a target of potassium-sparing diuretics and aldosterone antagonists, to 1.48 (95% CI, 0.03-74.76) per 10-mm Hg decrease in maternal SBP acting through *EDNRA*, a target of vasodilator antihypertensives. For maternal *ADRB*, a β-adrenoreceptor–blocking target, we estimated no differential risk of congenital malformation (estimated OR, 0.28 [95% CI, 0.02-4.71]) per 10-mm Hg decrease in SBP.

#### Offspring Developmental Score and Gestational Age

We estimated changes in offspring developmental score at 6 months (Figure 2F) ranging from −0.34 points (95% CI, −0.80 to 0.13 points) per 10-mm Hg decrease in maternal SBP acting through *ADRB1*, a target of β-adrenoceptor-blocking drugs, to 0.98 points (95% CI, 0.27-1.68 points) per 10-mm Hg decrease in maternal SBP acting through *SCNN1D*, a target of potassium-sparing diuretics and aldosterone antagonists. Changes in gestational age followed a similar pattern (Figure 2G), ranging from 0.53 days (95% CI, −1.55 to 0.50 days) per 10-mm Hg decrease in maternal SBP acting through *ADRB1* to 1.55 days (95% CI, −0.02 to 3.11 days) per 10-mm Hg decrease in maternal SBP acting through *SCNN1D*. For higher levels of maternal SBP acting through the *CACNB2* calcium channel blocker target, we estimated a change in gestational age of 3.99 days (95% CI, 0.02-7.96 days) per 10-mm Hg decrease in SBP.

#### Disorders of Pregnancy

Estimates for hypertensive disorders of pregnancy are shown in Figure 2I. The ORs ranged from 0.08 (95% CI, <0.01 to 0.76) per 10-mm Hg decrease in maternal SBP acting through *ADRB1*, a target of β-adrenoceptor-blocking drugs, to 2.79 (95% CI, 0.08-96.29) per 10-mm Hg decrease in maternal SBP acting through *EDNRA*, a target of vasodilator antihypertensives.

### Sensitivity Analysis

#### Paternal and Offspring Effect Estimates

Estimates for offspring outcomes per 10-mm Hg decrease in paternal SBP are displayed in eFigure 3 and eTable 5 in Supplement 1. We estimated a change in birth weight *z *score of −1.31 (95% CI, −2.2 to 0.42) and a change in head circumference of −1.63 cm (95% CI, −3.07 to −0.18 cm) per 10-mm Hg decrease in paternal SBP acting through *SCNN1D*, a target of potassium-sparing diuretics and aldosterone antagonist. We also estimated a change in offspring development score at 6 months of 0.28 points (95% CI, 0.03-0.52 points) per 10-mm Hg decrease in paternal SBP acting through *CACNB2*, a target of calcium channel blockers. Estimates for offspring outcomes per 10-mm Hg decrease in offspring SBP are displayed in eTable 6 in Supplement 1. We estimated a change in offspring birth weight *z* score of 0.72 (95% CI, −0.08 to 1.53) per 10-mm Hg decrease in SBP acting through the paternal *EDNRA* vasodilator antihypertensive target.

#### Instrument Strength

*F* statistics were calculated for the individual instruments and, when multiple instruments were available, were averaged across the drug class (eTable 7 in Supplement 1). All *F* statistics were greater than 10, and mean *F* statistics ranged between 33.06 (*SCNN1D* potassium-sparing diuretics and aldosterone antagonist target) and 90.32 (*CACNB2* calcium channel blocker target).

## Discussion

In this MR study, we investigated the potential causal relationship between SBP via maternal antihypertensive drug targets and early infant outcomes. We derived novel summary data for early infant outcomes using individual participant data from MoBa and, with publicly available summary data from the IEU OpenGWAS database for the exposure, applied intergenerational within-family MR. We found some evidence that higher levels of maternal SBP acting through drug targets for antihypertensives had a potential causal relationship with improved early infant outcomes. Our results for most outcomes suggest that many genetic drug targets, such as the *ADRB1* β-adrenoreceptor–blocking target, are unlikely to have large detrimental effects on infant outcomes. Furthermore, our negative control exposure, SBP via paternal antihypertensive drug targets, suggested that SBP via the *EDNRA* vasodilator antihypertensive target had similar potential effects on most offspring outcomes in mothers and fathers. This finding suggests that the potential causal relationships for this target are unlikely to be due to intrauterine effects. There was evidence that SBP via maternal and paternal antihypertensive drug targets had potentially differentially affected some offspring outcomes.

Few antihypertensives within our positive control analysis demonstrated a potential causal relationship with reduced risk of maternal hypertensive disorders of pregnancy. However, we had limited statistical power to detect effects on this positive control outcome. Furthermore, these instruments were not selected as antihypertensive drug targets using BP measured in pregnant women or as instruments for preeclampsia, where mechanisms through which they act may differ.

Our detected potential causal relationships could be explained by horizontal pleiotropy,^52^ which occurs when a genetic variant influences both the exposure (eg, intrauterine drug exposure) and the outcome (eg, perinatal birth weight *z* score) through pathways other than the drug target of interest, violating the exclusion restriction criteria. This bias can be toward or away from the null.^23,52^

To identify the potential pleiotropic pathways, we checked whether the instruments were associated with other determinants of perinatal outcomes. Within the GWAS catalog, rs1557765 (vasodilator antihypertensive targeting *KCNJ11*) and rs1801253 (β-adrenoceptor–blocking drugs targeting *ADRB1*) were associated with body mass index, a known determinant of macrosomia and large for gestational age.^53,54^ We did not find evidence of an association between these genetic drug targets and perinatal birth weight *z* scores for either parent; however, a reduction in birth weight *z* score was found in the offspring genetic drug targets (eTable 6 in Supplement 1). All other SNVs were found to be associated with BP-related measures, which suggests that pleiotropic effects may not have strongly influenced our results.

The drug target genetic variants may influence the offspring outcomes via direct inheritance. However, we adjusted for the offspring’s genetic variants, which should have controlled for direct genetic effects.^23^

Assortative mating creates associations for a number of heritable phenotypes between maternal and paternal genotypes.^55^ Typically, this inflates the estimated effect estimate through violation of the exclusion restriction criteria if there is assortment on the exposure of interest. However, mothers and fathers are unlikely to assort on protein levels or BP (which is typically not observed in younger populations). Thus, assortative mating was unlikely to have substantially impacted our analyses. Evidence from analysis of siblings suggests that biological traits (eg, C-reactive protein) are less likely to be biased by population or familial effects.^56^

Population stratification, the systematic difference in allele frequencies and phenotypes between subsets of populations, may also confound MR estimates.^57^ The estimated potential causal genetic-to-phenotypic effects may be spurious associations explained by ancestral differences.^57,58^ MoBa is a large, relatively homogenous European sample; therefore, it is unlikely that confounding due to population stratification greatly biased our findings.^36,59^ Additionally, we adjusted for the top 20 principal components to minimize confounding due to residual population stratification.^57,59^ Furthermore, population structure is likely to affect maternal and paternal variants equally and cannot explain differential parental findings between mothers and fathers.

We investigated the potential effects of SBP acting through antihypertensive drug targets known to affect proteins that these drugs target. We cannot be certain that these genetic proxies for drug exposure would have a comparable effect to taking the drug of interest. However, the mechanism of action is likely to be the same.

Mendelian randomization has been used to predict maternal-perinatal effects within the literature; however, these studies typically used summary-level data from unrelated individuals and did not link maternal exposure and perinatal outcomes or have genetic data from parent-offspring trios. A recent study implemented MR to investigate the safety of β-adrenoreceptor–blocking drugs and calcium channel blockers in pregnancy.^31^ These drug subclasses are typically the most prescribed antihypertensive drugs during pregnancy. Yet, there is still little evidence from RCTs to evaluate the risks and benefits of use during pregnancy.^60,61^ The MR study found evidence to suggest that genetically proxied β-adrenoreceptor blockers may reduce birth weight.^31^ This relationship has been demonstrated in observational studies^62,63^ and is of clinical concern for health care professionals and individuals that may become pregnant when using this medication. Furthermore, the MR study found that genetically proxied calcium channel blockers may reduce the risk of preeclampsia and eclampsia.^31^

We were able to address a limitation of the previous 2-sample MR study^31^ through the inclusion of the perinatal and paternal genotypes. Conditioning on the offspring genotype may induce collider bias. However, the inclusion of the paternal genotype likely mitigates this bias. Furthermore, we could exclude pleiotropic effects via the offspring and father. In contrast to the previous 2-sample MR study,^31^ we found some evidence that reduced maternal SBP via *ADRB1*, a target of β-adrenoceptor–blocking drugs, had a potential causal relationship with lower odds of gestational hypertension, though our estimated OR was implausible. Furthermore, we did not find evidence that reduced maternal SBP via *CACNB2*, a target of calcium channel blockers, potentially affected birth weight. Additionally, we used a measure of birth weight standardized for gestational age at birth; thus, our outcome provided a more accurate assessment of potential exposure effects on fetal growth, as the standardized measure accounts for variations in birth weight that occur at different stages of pregnancy relative to a measure of birth weight.

Two relevant RCTs^64,65^ compared differing diastolic BP targets controlled pharmacologically and the development of maternal hypertension. One was the Control of Hypertension in Pregnancy Study (CHIPS),^64^ which examined severe maternal hypertension with perinatal outcomes in a cohort of individuals with nonproteinuric preexisting or gestational hypertension or diastolic BP between 90 and 150 mm Hg seen at the clinic during 14 to 36 weeks’ gestation. The other RCT, the Chronic Hypertension and Pregnancy (CHAP) trial,^65^ examined the relationship between maternal hypertension treatment status and perinatal outcomes among pregnant individuals with mild chronic hypertension and gestational age less than 23 weeks.

The CHIPS and CHAP trials both established that less stringent target maternal BP caused more severe hypertension.^64,65^ Furthermore, the CHIPS trial found that severe hypertension was associated with adverse perinatal outcomes.^64^ Similarly, the CHAP trial found that participants who received pharmacologic treatment were at lower risk of adverse perinatal outcomes compared with those who did not receive treatment.^65^ The CHAP trial found that active treatment reduced risk of adverse perinatal outcomes.^65^ The CHIPS and CHAP trial results were not analyzed at the drug subclass level with an intervention of either labetalol (β-adrenoreceptor–blocking drug), nifedipine or amlodipine (calcium channel blockers), or methyldopa (centrally acting antihypertensive drug). Common outcomes across our study appeared to align with results from the CHIPS trial, as we estimated no differential perinatal risk associated with maternal genetic variant targets for β-adrenoreceptor–blocking drugs or calcium channel blockers and, thus, no evidence of potential fetal risk. We take caution in further comparison due to inherent study differences as we did not combine drug subclasses.

Observational analyses have suggested that maternal antihypertensive use is associated with lower risk of severe maternal hypertension, thus reducing the detrimental effects of the disease.^62,63^ However, associations between β-blockers and increased risk of offspring born small for gestational age were found. One study^62^ noted that the results may have been confounded by disease severity, and both concluded that further evidence is required due to inherent limitations of observational study designs.

We found little evidence for a potential causal relationship between maternal SBP acting through the drug targets and congenital malformation in this study. This is in agreement with the literature; for example, a meta-analysis of RCTs and observational studies found no evidence for increased odds of major congenital abnormalities when considering first-trimester β-adrenoreceptor–blocking drug use vs no use.^66^ Subsequent studies further controlling for maternal confounders also reported little evidence of congenital malformations following maternal use of calcium channel blockers, vasodilators, and diuretics.^16,67,68^

### Strengths and Limitations

Our study has several strengths. First, MoBa is a large, extensive, and detailed parent-offspring trio dataset. This enabled novel genetic investigation into potential intrauterine effects while controlling for genetic confounding and pleiotropy by including offspring and paternal genotype. Genotypic data were passed through a strict quality-control pipeline, and batch effects and principal components may have been adjusted for (eAppendix 5 in Supplement 1).^40^ Additionally, many perinatal outcomes of interest to this study originated from a national birth registry and, thus, were precisely measured with little missingness.

In this study, we estimated the potential effects of genetically perturbating specific antihypertensive drug targets. We were not able to assess the effects of taking a specific drug, which would represent the combined effects of all the drug’s targets, the dose, duration, indication timing, and so on. However, genetic evidence about the effects of each drug target can be informative about possible effects of perturbating each target. Second, MR effect estimates were interpreted as the potential effect of lifetime exposure.^69^ It is unlikely that maternal levels of these proteins would have large biological effects outside pregnancy. Third, we implemented 2-sample MR with nonoverlapping samples, which increased statistical power, alongside reducing the likelihood of the exaggeration of discovery results from the null relative to subsequent replication studies (ie, winner’s curse) and weak instrument bias.^70,71^ Additionally, in 2-sample MR, weak instrument bias is toward the null, avoiding false-positive findings.^19,71^ Fourth, our perinatal outcomes of interest were immediately after birth. Thus, it was temporally impossible for postnatal factors to have affected all but 1 of these outcomes and additional confounding bias was avoided. Fifth, for the drug subclasses in which we retained multiple genetic instruments, we performed IVW analysis to increase precision within estimates and provide greater certainty to our results. The use of multiple SNVs also increased the proportion of variance explained in the exposure by the instrument, increasing statistical power.^19^ Sixth, the instruments used in the SNV-exposure relationship were found to be sufficiently strong. Seventh, MR enables the exploration of a wider range of drugs. An RCT involving human participants would necessitate some degree of established safety profile. However, as MR does not involve active intervention or exposure beyond that of a typical pregnancy, we were not constrained to previously studied drugs.

This study also has limitations. Specifically, in our study, lifetime exposure to maternal genetic variants related to the intrauterine period for the offspring, with most study outcomes measured immediately at birth. Yet, genetic variants exert small lifetime effects relative to typical drug exposure (ie, larger over a specific period). Therefore, we anticipate that our estimated effect sizes may not directly equate to clinical results and encourage interpretation based on the potential direction of effect estimates, not the magnitude. Additionally, each individual genetic variant explains a small proportion of the variation. Thus, although the instruments were above the *F* statistic threshold, we may have had insufficient power to detect clinically meaningful effect sizes. However, we could discern conservative evidence of potential effects for some maternal genetically proxied drug subclasses. Drug target MR is not equivalent to estimating the impact of a pharmacologic intervention; rather, we focused on interpreting the estimated direction of effect estimates, not magnitude. Our results demonstrated many null findings, but the study was not powered to necessarily exclude small but clinically meaningful effects.

We could not perform standard MR sensitivity analyses, such as weighted median and weighted mode, to assess the exclusion restriction criterion as these require a larger number of SNVs for the exposure.^72^ This is a common limitation of drug target MR studies; however, this should be offset against the biological proximity of the genetic variants, which reduces the likelihood of pleiotropic effects.^73^ Additionally, we were unable to test for heterogeneity, such as calculating the Cochran *Q* statistic, due to the small number of SNVs in our analyses after all exclusions and restrictions were applied.^74^

We used genetic variation in mothers, fathers, and offspring to investigate the potential differential effects of reducing SBP via a range of antihypertensive drug targets on offspring outcomes. However, our results may reflect the potential effects of these targets that were not mediated via SBP (ie, horizontal pleiotropic effects). Furthermore, the genetic variants within a gene that proxy a protein drug target may influence many things related to the protein, including protein levels, structure, and function, so we could not be certain that we captured differences in protein levels alone.

This study required large-scale linked familial trio data to control for genetic confounding adequately and have sufficient power to detect potential effects. After exclusions and quality control, our sample size was reduced, which reduced statistical power. Furthermore, we could not investigate many drug subclasses that were initially identified. However, we had sufficient statistical power to detect effects on many outcomes and could exclude clinically meaningful effects for many negative control outcomes. There are currently few large datasets in which genetic information for trios and phenotypic information are available for replication. While the power of our study was naturally limited (which may have prevented inference for rare events and weak effect estimates), this study provides proof of concept and a framework for this approach to maternal-perinatal analyses, ideally via large-scale international consortia.

The MoBa study was subject to selection bias as recruitment into MoBa was contingent on a pregnancy reaching approximately 18 weeks; therefore, we were unable to assess the outcome of early pregnancy loss. Although their characteristics generally reflect the demographics in Norway, mothers in MoBa were older and had better health than the general population.^35,36,75^ This may limit the generalizability of our findings and introduce collider bias. However, selection was unlikely based on genetic variants for protein levels, which are typically unknown to participants. There were 918 mothers in MoBa (228 [0.8%] of this study’s cohort of trios) who reported antihypertensive use at any point within their pregnancy. This is unlikely to have substantially biased our results relative to an untreated population.

Many drugs have multiple targets. However, we could only assess targets for which a suitable instrument could be identified (ie, targets for which there were independent SNVs associated with SBP at genome-wide significance). Furthermore, we did not know the relative contribution of each drug target to each drug’s aggregate effect. Therefore, our instruments may not reflect the combined effect of all targets for a medication.

The genetic instruments were selected from a GWAS of SBP conducted in UK Biobank, which includes female and male participants aged 40 to 69 years. While instruments selected from this GWAS are likely to reflect the broad SBP phenotype, they may miss female-specific or time-dependent effects due to the inclusion of males and older individuals, respectively. Female-specific SNVs could be identified by using a GWAS conducted in female participants only. However, we were unable to find a publicly available GWAS for this purpose.^76^ Time-dependent effects are a known issue with MR.^77^

## Conclusions

Systematic evidence and guidance regarding the use of prescriptive drugs in pregnancy is severely lacking. In this MR study, our results suggest that reducing SBP via a range of antihypertensive drug targets is unlikely to have a potential causal relationship with adverse offspring perinatal outcomes that we investigated. We provided a framework for future investigations of the potential effects of intrauterine drug exposure on perinatal outcomes. This provides an additional source of evidence, which should be triangulated with other observational study designs and RCTs, when appropriate, to enhance the robustness of findings and inform clinical decision-making.

## Notes

Supplement 1. **eTable 1.** Nonexhaustive Review of the Literature Describing Adverse Neonatal Outcomes Following Exposure to the Indicated Drugs and Drug Subclasses **eTable 2.** Drug Substances, Corresponding Genes, and BNF Codes Used in the Instrument Selection Procedure **eTable 3.** Summary Statistics for Study Outcome Measures Within the Norwegian Mother, Father and Child Cohort Study **eTable 4.** MR Results for the Association of the Maternal Genetic Drug Protein Targets With Offspring Outcomes **eTable 5.** MR Results for the Association of the Paternal Genetic Drug Protein Targets With Offspring Outcomes **eTable 6.** MR Results for Association of the Offspring Genetic Drug Protein Targets with Offspring Outcomes **eTable 7.** Individual and Mean *F* Statistics for the Wald Ratio and IVW Estimates to Demonstrate Instrument Strength in the SNP-Exposure Association **eAppendix 1.** STROBE-MR Guidelines **eAppendix 2.** Sources Used to Determine Antihypertensives of Interest to This Study **eAppendix 3.** Derivation of the Prorated Developmental Score **eAppendix 4.** UK Biobank **eAppendix 5.** Quality Control of MoBa Data **eFigure 1.** Exclusion Flowchart Demonstrating the Cohort Derivation **eFigure 2.** Flowchart Describing the Instrument Derivation Procedure **eFigure 3.** Forest Plots of Estimated Associations Between the Paternal Genetic Drug Targets and Offspring Outcomes
eReferences

Supplement 2.
Data Sharing Statement
