# Supplementary material for: Genetic Insights Into Perinatal Outcomes of Maternal Antihypertensive Therapy During Pregnancy
Source: JAMA Netw Open. 2024 Aug 27;7(8):e2426234. doi: 10.1001/jamanetworkopen.2024.26234 (PMC11350473; doi:10.1001/jamanetworkopen.2024.26234)
Supplement: Supplement 1. — eTable 1. Nonexhaustive Review of the Literature Describing Adverse Neonatal Outcomes Following Exposure to the Indicated Drugs and Drug Subclasses eTable 2. Drug Substances, Corresponding Genes, and BNF Codes Used in the Instrument Selection Procedure eTable 3. Summary Statistics for Study Outcome Measures Within the Norwegian Mother, Father and Child Cohort Study eTable 4. MR Results for the Association of the Maternal Genetic Drug Protein Targets With Offspring Outcomes eTable 5. MR Results for the Association of the Paternal Genetic Drug Protein Targets With Offspring Outcomes eTable 6. MR Results for Association of the Offspring Genetic Drug Protein Targets with Offspring Outcomes eTable 7. Individual and Mean F Statistics for the Wald Ratio and IVW Estimates to Demonstrate Instrument Strength in the SNP-Exposure Association eAppendix 1. STROBE-MR Guidelines eAppendix 2. Sources Used to Determine Antihypertensives of Interest to This Study eAppendix 3. Derivation of the Prorated Developmental Score eAppendix 4. UK Biobank eAppendix 5. Quality Control of MoBa Data eFigure 1. Exclusion Flowchart Demonstrating the Cohort Derivation eFigure 2. Flowchart Describing the Instrument Derivation Procedure eFigure 3. Forest Plots of Estimated Associations Between the Paternal Genetic Drug Targets and Offspring Outcomes eReferences [file jamanetwopen-e2426234-s001.pdf]

Supplementary Online Content

Barry CJS, Walker VM, Burden C, Havdahl A, Davies NM. Genetic insights into perinatal outcomes of maternal antihypertensive drugs during pregnancy. *JAMA Netw Open*. 2024;7(8):e2426234. doi:10.1001/jamanetworkopen.2024.26234

eTable 1. Nonexhaustive Review of the Literature Describing Adverse Neonatal Outcomes Following Exposure to the Indicated Drugs and Drug Subclasses
eTable 2. Drug Substances, Corresponding Genes, and BNF Codes Used in the Instrument Selection Procedure
eTable 3. Summary Statistics for Study Outcome Measures Within the Norwegian Mother, Father and Child Cohort Study
eTable 4. MR Results for the Association of the Maternal Genetic Drug Protein Targets With Offspring Outcomes
eTable 5. MR Results for the Association of the Paternal Genetic Drug Protein Targets With Offspring Outcomes
eTable 6. MR Results for Association of the Offspring Genetic Drug Protein Targets with Offspring Outcomes
eTable 7. Individual and Mean *F* Statistics for the Wald Ratio and IVW Estimates to Demonstrate Instrument Strength in the SNV-Exposure Association
eAppendix 1. STROBE-MR Guidelines
eAppendix 2. Sources Used to Determine Antihypertensives of Interest to This Study
eAppendix 3. Derivation of the Prorated Developmental Score
eAppendix 4. UK Biobank
eAppendix 5. Quality Control of MoBa Data
eFigure 1. Exclusion Flowchart Demonstrating the Cohort Derivation
eFigure 2. Flowchart Describing the Instrument Derivation Procedure
eFigure 3. Forest Plots of Estimated Associations Between the Paternal Genetic Drug Targets and Offspring Outcomes
eReferences

This supplementary material has been provided by the authors to give readers additional information about their work.

© 2024 Barry CJS et al. *JAMA Network Open*.

eTable 1: A non-exhaustive review of the literature describing adverse neonatal outcomes following exposure to the indicated drugs/drug subclasses. Note that this includes a range of retrospective, case series, case studies, and observational studies that may be biased due to confounding.

**Drug/drug subclass**	**Adverse neonatal outcome**
Beta-adrenergic receptor antagonists	Impaired foetal growth, neonatal hypoglycaemia (1–4)
Alpha adrenergic blocking agents (ARB)	Congenital malformations, fetopathy (5–7)
Angiotensin-converting enzyme (ACE) inhibitors	Fetopathy, congenital malformations, miscarriage (5–8)
Calcium channel blockers (CCB)	Seizures (3,8)
Vasodilators	Hypertrichosis, low Apgar score, placental abruption (9,10)

© 2024 Barry CJS et al. *JAMA Network Open*.

eTable 2: A table of the drug substances, corresponding genes and BNF codes used in the instrument selection procedure.

**Drug substance**	**DrugBank ID**	**Gene**	**BNF Code**
Acebutolol	DB01193	ADRB1	204000
Aliskiren	DB09026	REN	205053
Ambrisentan	DB06403	EDNRA	205010
Amiloride	DB00594	SCNN1A, SCNN1D, SCNN1G, SCNN1B	202030
Amlodipine	DB00381	CACNA1C, CACNA1I	206020
Atenolol	DB00335	ADRB1	204000
Azilsartan Medoxomil	DB08822	AGTR1	205052
Bendroflumethiazide	DB00436	SLC12A3, KCNMA1	202010
Betaxolol	DB00195	ADRB1	204000
Bisoprolol	DB00612	ADRB1	204000
Bosentan	DB00559	EDNRA, EDNRB	205010
Bumetanide	DB00887	SLC12A4, SLC12A1, SLC12A2, SLC12A5	202020
Candesartan	DB13919	AGTR1	205052
Captopril	DB01197	ACE	205051
Carvedilol	DB01136	ADRB1, ADRA1A, ADRA1B, ADRA1D	20400080
Celiprolol	DB04846	ADRB2, ADRB1	20400060
Chlorothiazide	DB00880	CA1, CA2, SLC12A3	202010
Chlortalidone	DB00310	CA1, SLC12A1	202010
Cilazapril	DB01340	ACE	205051
Clonidine	DB00575	ADRA2B, ADRA2C, ADRA2A	205020
Debrisoquine	DB04840	SLC6A2	205030
Diazoxide	DB01119	KCNJ11, CA2, CA1	205010
Diltiazem	DB00343	CACNG1, CACNA1C	206020
Doxazosin	DB00590	ADRA1D, ADRA1A	205040
Enalapril	DB00584	ACE	205051
Eplerenone	DB00700	NR3C2	202030
Eprosartan	DB00876	AGTR1	205052
Felodipine	DB01023	CACNA1S, CACNB2, CACNA2D1, CACNA1D, CACNA1C	206020
Finerenone	DB16165	NR3C2	202030
Fosinopril	DB00492	ACE	205051
Furosemide	DB00695	SLC12A1	202020
Guanethidine	DB01170	SLC6A2	205030
Guanfacine	DB01018	ADRA2A	205020
Hydralazine	DB01275	AOC3	205010
Hydrochlorothiazide	DB00999	KCNMA1, SLC12A3	202010

© 2024 Barry CJS et al. *JAMA Network Open*.

Iloprost	DB01088	PTGIR, PTGER1	205010
Indapamide	DB00808	SLC12A3	202010
Irbesartan	DB01029	AGTR1	205052
Isradipine	DB00270	CACNA1H, CACNA2D1, CACNA1D, CACNB2, CACNA1S, CACNA2D2, CACNA1C	206020
Labetalol	DB00598	ADRB1, ADRB2, ADRA1B, ADRA1A, ADRA1D	204000
Lacidipine	DB09236	CACNB1, CACNB2, CACNB3, CACNA1S, CACNA1A, CACNA1D, CACNA1F, CACNA1C, CACNB4	206020
Lercanidipine	DB00528	CACNG1	206020
Lisinopril	DB00722	ACE	205051
Losartan	DB00678	AGTR1	205052
Macitentan	DB08932	EDNRA	205010
Meprobamate	DB00371	GABRG1, GABRA5, GABRB2, GABRG3, GABRA3, GABRA2, GABRA4, GABRG2, GABRA1, GABRQ, GABRB1, GABRE, GABRP, GABRA6, GABRB3, GABRD	401020
Methyldopa	DB00968	DDC, ADRA2A	205020
Metirosine	DB00765	TH	
Metolazone	DB00524	SLC12A3	202010
Metoprolol	DB00264	ADRB1	204000
Minoxidil	DB00350	KCNJ1	205010
Moexipril	DB00691	ACE	205051
Moxonidine	DB09242	NISCH, ADRA2A	205020
Nadolol	DB01203	ADRB1	204000
Nebivolol	DB04861	ADRB1	204000
Nicardipine	DB00622	CACNA1C, CACNB2, CACNA1D, CACNA2D1	206020
Nifedipine	DB01115	CACNA1C, CACNB2, CACNA1D	206020
Nimodipine	DB00393	CACNA1F, CACNA1C, CACNB1, CACNB3, CACNB2, CACNA1S, CACNA1D, CACNB4	206020
Nisoldipine	DB00401	CACNB2, CACNA1S, CACNA2D1, CACNA1D, CACNA1C	206020
Oxprenolol	DB01580	ADRB1	204000
Perhexiline	DB01074	CPT2, CPT1A	N/A
Phenoxybenzamine	DB00925	ADRA1A, ADRA2A	205040
Phentolamine	DB00692	ADRA1A, ADRA2A	205040
Pindolol	DB00960	ADRB1, ADRB2	204000
Polythiazide	DB01324	SLC12A3	202010
Prazosin	DB00457	ADRA1A, ADRA1B, ADRA1D	205040
Propranolol	DB00571	ADRB1	204000

© 2024 Barry CJS et al. *JAMA Network Open*.

Quinapril	DB00881	ACE	205051
Ramipril	DB00178	ACE	205051
Riociguat	DB08931	GUCY1A2	205010
Sildenafil	DB00203	PDE5A	205010
Sitaxentan	DB06268	EDNRA	205010
Sotalol	DB00489	KCNH2, ADRB2, ADRB1	204000
Spironolactone	DB00421	NR3C2	202030
Tadalafil	DB00820	PDE5A	704050
Tamsulosin	DB00706	ADRA1A	704010
Telmisartan	DB00966	AGTR1, PPARG	205052
Terazosin	DB01162	ADRA1B, ADRA1A, ADRA1D, TGFB1	205040
Timolol	DB00373	ADRB1, ADRB2	204000
Torasemide	DB00214	SLC12A1, SLC12A2	202020
Trandolapril	DB00519	ACE	205051
Triamterene	DB00384	SCNN1G, SCNN1B, SCNN1A	202030
Valsartan	DB00177	AGTR1	205052
Verapamil	DB00661	CACNA1C	206020
Vericiguat	DB15456	GUCY1B1	205010
Olmesartan	DB00275	AGTR1	205052
Perindopril	DB00790	ACE	205051

BNF; British National Formulary

© 2024 Barry CJS et al. *JAMA Network Open*.

eTable 3: Summary statistics for the study outcome measures within the Norwegian Mother, Father and Child Cohort Study (MoBa).

**Variable**	**Mean (SD) or count (%)**	**NA (N)**
Hypertensive disorders of pregnancy		0
Yes	1703 (5.70)	
No	28196 (94.3)	
Gestational age	279.32 (12.18)	128
Head circumference (cm)	35.28 (1.61)	543
Apgar score at 1 minute	8.69 (1.13)	59
Apgar score at 5 minutes	9.43 (0.79)	58
Birth length (cm)	50.36 (2.4)	1150
Developmental score	9.45 (0.73)	4400
Birthweight for gestational age z-score	0.12 (0.98)	127
Congenital malformations		0
Yes	1,350 (4.52)	
No	28,499 (95.48)	

SD; standard deviation

© 2024 Barry CJS et al. *JAMA Network Open*.

eTable 4: The MR results for the effect of the maternal genetic drug protein targets on offspring outcomes.

Outcome	Beta coefficient (95% CI)	Standard error	Pvalue	Drug subclass	MR method	rsID
Gestational age (days)	3.99 (0.0249, 7.96)	3.92	0.048	Calcium-channel blockers targeting CACNB2 (3 SNVs)	IVW	rs10764331 rs12258967 rs3821843
Birthweight for gestational age z-score	-0.195 (-0.496, 0.107)	0.298	0.205	Calcium-channel blockers targeting CACNB2 (3 SNVs)	IVW	rs10764331 rs12258967 rs3821843
Birth length (cm)	-0.477 (-1.74, 0.782)	1.24	0.458	Calcium-channel blockers targeting CACNB2 (3 SNVs)	IVW	rs10764331 rs12258967 rs3821843
Head circumference (cm)	0.136 (-0.352, 0.625)	0.483	0.584	Calcium-channel blockers targeting CACNB2 (3 SNVs)	IVW	rs10764331 rs12258967 rs3821843
Apgar score, 1 minute	0.0917 (-0.256, 0.439)	0.344	0.605	Calcium-channel blockers targeting CACNB2 (3 SNVs)	IVW	rs10764331 rs12258967 rs3821843
Apgar score, 5 minutes	0.0538 (-0.187, 0.294)	0.237	0.661	Calcium-channel blockers targeting CACNB2 (3 SNVs)	IVW	rs10764331 rs12258967 rs3821843
Hypertensive disorders of pregnancy	0.610 (0.159, 2.34)	1.33	0.470	Calcium-channel blockers targeting CACNB2 (3 SNVs)	IVW	rs10764331 rs12258967 rs3821843

© 2024 Barry CJS et al. *JAMA Network Open*.

Developmental score	0.158 (-0.0837, 0.399)	0.239	0.201	Calcium-channel blockers targeting CACNB2 (3 SNVs)	IVW	rs10764331 rs12258967 rs3821843
Congenital malformation	2.08 (0.480, 9.05)	1.45	0.327	Calcium-channel blockers targeting CACNB2 (3 SNVs)	IVW	rs10764331 rs12258967 rs3821843
Gestational age (days)	10.7 (-0.0767, 21.5)	10.7	0.0516	Potassium-sparing diuretics and aldosterone antagonists targeting SCNN1D (1 SNV)	Wald ratio	rs1262894
Gestational age (days)	7.34 (-2.55, 17.2)	9.77	0.146	Vasodilator Antihypertensive Drugs targeting EDNRA (1 SNV)	Wald ratio	rs13143677
Gestational age (days)	1.03 (-8.32, 10.4)	9.24	0.830	Vasodilator Antihypertensive Drugs targeting KCNJ11 (1 SNV)	Wald ratio	rs1557765
Gestational age (days)	-3.54 (-10.6, 3.55)	7.01	0.328	Beta-adrenoreceptor blocking drugs targeting ADRB1 (1 SNV)	Wald ratio	rs1801253
Birthweight for gestational age z-score	-0.111 (-0.986, 0.764)	0.865	0.804	Potassium-sparing diuretics and aldosterone antagonists targeting SCNN1D (1 SNV)	Wald ratio	rs1262894
Birthweight for	0.711 (-0.0899, **1.51)**	0.792	0.0819	Vasodilator Antihypertensive Drugs	Wald ratio	rs13143677

© 2024 Barry CJS et al. *JAMA Network Open*.

gestational age z-score				targeting EDNRA (1 SNV)		
Birthweight for gestational age z-score	-0.685 (-1.44, 0.0732)	0.750	0.0766	Vasodilator Antihypertensive Drugs targeting KCNJ11 (1 SNV)	Wald ratio	rs1557765
Birthweight for gestational age z-score	0.353 (-0.229, 0.929)	0.569	0.229	Beta-adrenoreceptor blocking drugs targeting ADRB1 (1 SNV)	Wald ratio	rs1801253
Birth length (cm)	1.07 (-1.07, 3.21)	2.12	0.326	Potassium-sparing diuretics and aldosterone antagonists targeting SCNN1D (1 SNV)	Wald ratio	rs1262894
Birth length (cm)	2.03 (0.0646, 3.99)	1.94	0.0429	Vasodilator Antihypertensive Drugs targeting EDNRA (1 SNV)	Wald ratio	rs13143677
Birth length (cm)	-0.303 (-2.16, 1.55)	1.83	0.748	Vasodilator Antihypertensive Drugs targeting KCNJ11 (1 SNV)	Wald ratio	rs1557765
Birth length (cm)	-0.146 (-1.55, 1.26)	1.39	0.839	Beta-adrenoreceptor blocking drugs targeting ADRB1 (1 SNV)	Wald ratio	rs1801253
Head circumference (cm)	0.945 (-0.474, 2.36)	1.40	0.192	Potassium-sparing diuretics and aldosterone antagonists targeting	Wald ratio	rs1262894

				SCNN1D (1 SNV)		
Head circumference (cm)	0.732 (-0.569, 2.03)	1.29	0.270	Vasodilator Antihypertensive Drugs targeting EDNRA (1 SNV)	Wald ratio	rs13143677
Head circumference (cm)	-0.753 (-1.98, 0.476)	1.22	0.230	Vasodilator Antihypertensive Drugs targeting KCNJ11 (1 SNV)	Wald ratio	rs1557765
Head circumference (cm)	-0.0144 (-0.947, 0.918)	0.922	0.976	Beta-adrenoreceptor blocking drugs targeting ADRB1 (1 SNV)	Wald ratio	rs1801253
Apgar score, 1 minute	0.466 (-0.546, 1.48)	1.00	0.367	Potassium-sparing diuretics and aldosterone antagonists targeting SCNN1D (1 SNV)	Wald ratio	rs1262894
Apgar score, 1 minute	-0.263 (-1.19, 0.667)	0.920	0.580	Vasodilator Antihypertensive Drugs targeting EDNRA (1 SNV)	Wald ratio	rs13143677
Apgar score, 1 minute	0.389 (-0.488, 1.27)	0.868	0.385	Vasodilator Antihypertensive Drugs targeting KCNJ11 (1 SNV)	Wald ratio	rs1557765
Apgar score, 1 minute	0.415 (-0.252, 1.08)	0.659	0.222	Beta-adrenoreceptor blocking drugs targeting ADRB1 (1 SNV)	Wald ratio	rs1801253

© 2024 Barry CJS et al. *JAMA Network Open*.

Apgar score, 5 minutes	0.352 (-0.350, 1.05)	0.694	0.326	Potassium-sparing diuretics and aldosterone antagonists targeting SCNN1D (1 SNV)	Wald ratio	rs1262894
Apgar score, 5 minutes	-0.498 (-1.14, 0.147)	0.637	0.130	Vasodilator Antihypertensive Drugs targeting EDNRA (1 SNV)	Wald ratio	rs13143677
Apgar score, 5 minutes	0.437 (-0.173, 1.05)	0.603	0.160	Vasodilator Antihypertensive Drugs targeting KCNJ11 (1 SNV)	Wald ratio	rs1557765
Apgar score, 5 minutes	0.0374 (-0.425, 0.500)	0.457	0.874	Beta-adrenoreceptor blocking drugs targeting ADRB1 (1 SNV)	Wald ratio	rs1801253
Hypertensive disorders of pregnancy	0.08 (0, 4.35)	3.96	0.21	Potassium-sparing diuretics and aldosterone antagonists targeting SCNN1D (1 SNV)	Wald ratio	rs1262894
Hypertensive disorders of pregnancy	2.79 (0.0809, 96.3)	3.50	0.570	Vasodilator Antihypertensive Drugs targeting EDNRA (1 SNV)	Wald ratio	rs13143677
Hypertensive disorders of pregnancy	1.42 (0.0491, 41.2)	3.33	0.838	Vasodilator Antihypertensive Drugs targeting KCNJ11 (1 SNV)	Wald ratio	rs1557765

© 2024 Barry CJS et al. *JAMA Network Open*.

Hypertensive disorders of pregnancy	0.0570 (0.00425, 0.765)	2.57	0.0306	Beta-adrenoreceptor blocking drugs targeting ADRB1 (1 SNV)	Wald ratio	rs1801253
Developmental score	0.950 (0.250, 1.65)	0.69	0.01	Potassium-sparing diuretics and aldosterone antagonists targeting SCNN1D (1 SNV)	Wald ratio	rs1262894
Developmental score	-0.0910 (-0.737, 0.555)	0.639	0.783	Vasodilator Antihypertensive Drugs targeting EDNRA (1 SNV)	Wald ratio	rs13143677
Developmental score	0.166 (-0.442, 0.774)	0.601	0.592	Vasodilator Antihypertensive Drugs targeting KCNJ11 (1 SNV)	Wald ratio	rs1557765
Developmental score	-0.324 (-0.784, 0.136)	0.455	0.167	Beta-adrenoreceptor blocking drugs targeting ADRB1 (1 SNV)	Wald ratio	rs1801253
Congenital malformation	0.120 (0.00139, 10.4)	4.41	0.352	Potassium-sparing Diuretics and Aldosterone Antagonists targeting SCNN1D (1 SNV)	Wald ratio	rs1262894
Congenital malformation	1.48 (0.0293, 74.8)	3.88	0.845	Vasodilator Antihypertensive Drugs targeting EDNRA (1 SNV)	Wald ratio	rs13143677

© 2024 Barry CJS et al. *JAMA Network Open*.

Congenital malformation	0.518 (0.0122, 22.0)	3.71	0.731	Vasodilator Antihypertensive Drugs targeting KCNJ11 (1 SNV)	Wald ratio	rs1557765
Congenital malformation	0.278 (0.0165, 4.71)	2.80	0.376	Beta-adrenoceptor Blocking drugs targeting ADRB1 (1 SNV)	Wald ratio	rs1801253

© 2024 Barry CJS et al. *JAMA Network Open*.

eTable 5: The MR results for the association of the paternal genetic drug protein targets with offspring outcomes.

**Outcome**	**Beta coefficient (95% CI)**	**Standard error**	**Pvalue**	**Drug subclass**	**MR method**	**rsID**
Gestational age (days)	-2.20 (-9.37, 4.98)	7.10	0.548	Beta-adrenoreceptor blocking drugs targeting ADRB1 (1 SNV)	Wald ratio	rs1801253
Birthweight for gestational age z-score	-0.0750 (-0.657, 0.507)	0.576	0.800	Beta-adrenoreceptor blocking drugs targeting ADRB1 (1 SNV)	Wald ratio	rs1801253
Birth length (cm)	-0.549 (-1.97, 0.875)	1.41	0.450	Beta-adrenoreceptor blocking drugs targeting ADRB1 (1 SNV)	Wald ratio	rs1801253
Head circumference (cm)	-0.254 (-1.20, 0.691)	0.934	0.599	Beta-adrenoreceptor blocking drugs targeting ADRB1 (1 SNV)	Wald ratio	rs1801253
Apgar score, 1 minute	0.378 (-0.297, 1.05)	0.667	0.272	Beta-adrenoreceptor blocking drugs targeting ADRB1 (1 SNV)	Wald ratio	rs1801253
Apgar score, 5 minutes	0.152 (-0.316, 0.621)	0.463	0.524	Beta-adrenoreceptor blocking drugs targeting ADRB1 (1 SNV)	Wald ratio	rs1801253

© 2024 Barry CJS et al. *JAMA Network Open*.

Hypertensive disorders of pregnancy	0.292 (0.0217, 3.93)	2.57	0.354	Beta-adrenoreceptor blocking drugs targeting ADRB1 (1 SNV)	Wald ratio	rs1801253
Developmental score	-0.0487 (-0.516, 0.418)	0.462	0.838	Beta-adrenoreceptor blocking drugs targeting ADRB1 (1 SNV)	Wald ratio	rs1801253
Gestational age (days)	-8.04 (-19.0, 2.93)	10.9	0.151	Potassium-sparing diuretics and aldosterone antagonists targeting SCNN1D (1 SNV)	Wald ratio	rs1262894
Birthweight for gestational age z-score	-1.31 (-2.20, -0.418)	0.879	>0.001	Potassium-sparing diuretics and aldosterone antagonists targeting SCNN1D (1 SNV)	Wald ratio	rs1262894
Birth length (cm)	-1.73 (-3.898, 0.44)	2.14	0.118	Potassium-sparing diuretics and aldosterone antagonists targeting SCNN1D (1 SNV)	Wald ratio	rs1262894
Head circumference (cm)	-1.63 (-3.07, -0.185)	1.43	0.0270	Potassium-sparing diuretics and aldosterone antagonists targeting SCNN1D (1 SNV)	Wald ratio	rs1262894
Apgar score, 1 minute	-0.734 (-1.76, 0.293)	1.02	0.161	Potassium-sparing diuretics and aldosterone	Wald ratio	rs1262894

© 2024 Barry CJS et al. *JAMA Network Open*.

				antagonists targeting SCNN1D (1 SNV)		
Apgar score, 5 minutes	-0.485 (-1.20, 0.227)	0.704	0.182	Potassium-sparing diuretics and aldosterone antagonists targeting SCNN1D (1 SNV)	Wald ratio	rs1262894
Hypertensive disorders of pregnancy	3.30 (0.0665, 164)	3.86	0.549	Potassium-sparing diuretics and aldosterone antagonists targeting SCNN1D (1 SNV)	Wald ratio	rs1262894
Developmental score	0.110 (-0.603, 0.822)	0.705	0.763	Potassium-sparing diuretics and aldosterone antagonists targeting SCNN1D (1 SNV)	Wald ratio	rs1262894
Gestational age (days)	3.83 (-6.09, 13.8)	9.81	0.449	Vasodilator Antihypertensive Drugs targeting EDNRA (1 SNV)	Wald ratio	rs13143677
Birthweight for gestational age z-score	0.724 (-0.0795, 1.53)	0.794	0.0774	Vasodilator Antihypertensive Drugs targeting EDNRA (1 SNV)	Wald ratio	rs13143677
Birth length (cm)	0.545 (-1.42, 2.51)	1.94	0.587	Vasodilator Antihypertensive Drugs targeting EDNRA (1 SNV)	Wald ratio	rs13143677
Head circumference (cm)	0.792 (-0.513, 2.10)	1.29	0.234	Vasodilator Antihypertensive Drugs targeting	Wald ratio	rs13143677

© 2024 Barry CJS et al. *JAMA Network Open*.

				EDNRA (1 SNV)		
Apgar score, 1 minute	-0.222 (-1.16, 0.711)	0.923	0.641	Vasodilator Antihypertensive Drugs targeting EDNRA (1 SNV)	Wald ratio	rs13143677
Apgar score, 5 minutes	-0.460 (-1.11, 0.187)	0.640	0.164	Vasodilator Antihypertensive Drugs targeting EDNRA (1 SNV)	Wald ratio	rs13143677
Hypertensive disorders of pregnancy	0.869 (0.0253, 29.8)	3.50	0.938	Vasodilator Antihypertensive Drugs targeting EDNRA (1 SNV)	Wald ratio	rs13143677
Developmental score	-0.510 (-1.16, 0.138)	0.641	0.123	Vasodilator Antihypertensive Drugs targeting EDNRA (1 SNV)	Wald ratio	rs13143677
Gestational age (days)	-3.75 (-13.1, 5.64)	9.29	0.434	Vasodilator Antihypertensive Drugs targeting KCNJ11 (1 SNV)	Wald ratio	rs1557765
Birthweight for gestational age z-score	-0.0365 (-0.799, 0.726)	0.754	0.925	Vasodilator Antihypertensive Drugs targeting KCNJ11 (1 SNV)	Wald ratio	rs1557765
Birth length (cm)	0.405 (-1.46, 2.27)	1.84	0.670	Vasodilator Antihypertensive Drugs targeting KCNJ11 (1 SNV)	Wald ratio	rs1557765
Head circumference (cm)	-1.04 (-2.28, 0.193)	1.22	0.0982	Vasodilator Antihypertensive Drugs targeting	Wald ratio	rs1557765

© 2024 Barry CJS et al. *JAMA Network Open*.

				KCNJ11 (1 SNV)		
Apgar score, 1 minute	0.341 (-0.541, 1.22)	0.872	0.449	Vasodilator Antihypertensive Drugs targeting KCNJ11 (1 SNV)	Wald ratio	rs1557765
Apgar score, 5 minutes	0.100 (-0.513, 0.713)	0.606	0.749	Vasodilator Antihypertensive Drugs targeting KCNJ11 (1 SNV)	Wald ratio	rs1557765
Hypertensive disorders of pregnancy	3.13 (0.105, 92.8)	3.35	0.510	Vasodilator Antihypertensive Drugs targeting KCNJ11 (1 SNV)	Wald ratio	rs1557765
Developmental score	-0.212 (-0.824, 0.400)	0.605	0.496	Vasodilator Antihypertensive Drugs targeting KCNJ11 (1 SNV)	Wald ratio	rs1557765
Gestational age (days)	3.16 (-3.03, 9.35)	6.12	0.317	Calcium-channel blockers targeting CACNB2 (3 SNVs)	IVW	rs10764331 rs12258967 rs3821843
Birthweight for gestational age z-score	-0.0923 (-0.399, 0.215)	0.304	0.556	Calcium-channel blockers targeting CACNB2 (3 SNVs)	IVW	rs10764331 rs12258967 rs3821843
Birth length (cm)	-0.126 (-0.865, 0.614)	0.731	0.739	Calcium-channel blockers targeting CACNB2 (3 SNVs)	IVW	rs10764331 rs12258967 rs3821843
Head circumference (cm)	0.0426 (-0.448, 0.533)	0.485	0.865	Calcium-channel blockers targeting	IVW	rs10764331 rs12258967 rs3821843

© 2024 Barry CJS et al. *JAMA Network Open*.

				CACNB2 (3 SNVs)		
Apgar score, 1 minute	-0.0295 (-0.379, 0.319)	0.345	0.868	Calcium-channel blockers targeting CACNB2 (3 SNVs)	IVW	rs10764331 rs12258967 rs3821843
Apgar score, 5 minutes	-0.0929 (-0.334, 0.148)	0.239	0.451	Calcium-channel blockers targeting CACNB2 (3 SNVs)	IVW	rs10764331 rs12258967 rs3821843
Hypertensive disorders of pregnancy	1.16 (0.0827, 16.4)	2.62	0.910	Calcium-channel blockers targeting CACNB2 (3 SNVs)	IVW	rs10764331 rs12258967 rs3821843
Developmental score	0.278 (0.0356, 0.520)	0.240	0.0246	Calcium-channel blockers targeting CACNB2 (3 SNVs)	IVW	rs10764331 rs12258967 rs3821843
Congenital malformation	3.58 (0.542, 23.6)	1.87	0.186	Calcium-channel blockers targeting CACNB2 (3 SNVs)	IVW	rs10764331 rs12258967 rs3821843
Congenital malformation	0.202 (0.00225, 18.2)	4.45	0.486	Potassium-sparing Diuretics and Aldosterone Antagonists targeting SCNN1D (1 SNV)	Wald ratio	rs1262894
Congenital malformation	0.422 (0.00839, 21.2)	3.87	0.666	Vasodilator Antihypertensive Drugs targeting EDNRA (1 SNV)	Wald ratio	rs13143677
Congenital malformation	5.77 (0.132, 251)	3.73	0.363	Vasodilator Antihypertensive Drugs	Wald ratio	rs1557765

© 2024 Barry CJS et al. *JAMA Network Open*.

				targeting KCNJ11 (1 SNV)		
Congenital malformation	0.378 (0.0217, 6.57)	2.82	0.504	Beta-adrenoceptor Blocking drugs targeting ADRB1 (1 SNV)	Wald ratio	rs1801253

© 2024 Barry CJS et al. *JAMA Network Open*.

eTable 6: The MR results for the assocation of the offspring genetic drug protein targets with offspring outcomes. Note estimates are from SBP instruments measured in adulthood.

**Outcome**	**Beta coefficient (95% CI)**	**Standard error**	**Pvalue**	**Drug subclass**	**MR method**	**rsID**
Gestational age (days)	8.94 (0.679, 17.2)	8.17	0.0339	Beta-adrenoreceptor blocking drugs targeting ADRB1 (1 SNV)	Wald ratio	rs1801253
Birthweight for gestational age z-score	-1.35 (-2.02, -0.675)	0.663	<0.001	Beta-adrenoreceptor blocking drugs targeting ADRB1 (1 SNV)	Wald ratio	rs1801253
Birth length (cm)	-0.975 (-2.61, 0.662)	1.62	0.243	Beta-adrenoreceptor blocking drugs targeting ADRB1 (1 SNV)	Wald ratio	rs1801253
Head circumference (cm)	-0.773 (-1.86, 0.313)	1.07	0.163	Beta-adrenoreceptor blocking drugs targeting ADRB1 (1 SNV)	Wald ratio	rs1801253
Apgar score, 1 minute	-0.507 (-1.28, 0.270)	0.767	0.201	Beta-adrenoreceptor blocking drugs targeting ADRB1 (1 SNV)	Wald ratio	rs1801253
Apgar score, 5 minutes	-0.335 (-0.873, 0.224)	0.533	0.224	Beta-adrenoreceptor blocking drugs targeting ADRB1 (1 SNV)	Wald ratio	rs1801253
Hypertensive disorders of pregnancy	0.434 (0.0213, 8.84)	2.98	0.587	Beta-adrenoreceptor blocking drugs targeting ADRB1 (1 SNV)	Wald ratio	rs1801253

© 2024 Barry CJS et al. *JAMA Network Open*.

Gestational age (days)	-1.34 (-13.8, 11.2)	12.4	0.834	Potassium-sparing diuretics and aldosterone antagonists targeting SCNN1D (1 SNV)	Wald ratio	rs1262894
Birthweight for gestational age z-score	0.955 (-0.0574, 1.97)	1.00	0.0645	Potassium-sparing diuretics and aldosterone antagonists targeting SCNN1D (1 SNV)	Wald ratio	rs1262894
Birth length (cm)	0.184 (-2.29, 2.66)	2.44	0.884	Potassium-sparing diuretics and aldosterone antagonists targeting SCNN1D (1 SNV)	Wald ratio	rs1262894
Head circumference (cm)	0.586 (-1.06, 2.23)	1.62	0.484	Potassium-sparing diuretics and aldosterone antagonists targeting SCNN1D (1 SNV)	Wald ratio	rs1262894
Apgar score, 1 minute	0.131 (-1.04, 1.30)	1.16	0.827	Potassium-sparing diuretics and aldosterone antagonists targeting SCNN1D (1 SNV)	Wald ratio	rs1262894
Apgar score, 5 minutes	-0.226 (-1.04, 0.586)	0.803	0.586	Potassium-sparing diuretics and aldosterone antagonists targeting SCNN1D (1 SNV)	Wald ratio	rs1262894

© 2024 Barry CJS et al. *JAMA Network Open*.

Hypertensive disorders of pregnancy	0.0512 (0.000510, 5.14)	4.56	0.206	Potassium-sparing diuretics and aldosterone antagonists targeting SCNN1D (1 SNV)	Wald ratio	rs1262894
Gestational age (days)	-6.07 (-17.4, 5.26)	11.2	0.294	Vasodilator Antihypertensive Drugs targeting EDNRA (1 SNV)	Wald ratio	rs13143677
Birthweight for gestational age z-score	-0.380 (-1.30, 0.534)	0.910	0.412	Vasodilator Antihypertensive Drugs targeting EDNRA (1 SNV)	Wald ratio	rs13143677
Birth length (cm)	-0.910 (-3.15, 1.34)	2.22	0.430	Vasodilator Antihypertensive Drugs targeting EDNRA (1 SNV)	Wald ratio	rs13143677
Head circumference (cm)	-0.402 (-1.89, 1.09)	1.47	0.597	Vasodilator Antihypertensive Drugs targeting EDNRA (1 SNV)	Wald ratio	rs13143677
Apgar score, 1 minute	0.0124 (-1.05, 1.08)	1.05	0.982	Vasodilator Antihypertensive Drugs targeting EDNRA (1 SNV)	Wald ratio	rs13143677
Apgar score, 5 minutes	0.482 (-0.257, 1.22)	0.731	0.201	Vasodilator Antihypertensive Drugs targeting EDNRA (1 SNV)	Wald ratio	rs13143677
Hypertensive disorders of pregnancy	0.0287 (0.000513, 1.60)	3.97	0.0835	Vasodilator Antihypertensive Drugs targeting EDNRA (1 SNV)	Wald ratio	rs13143677

© 2024 Barry CJS et al. *JAMA Network Open*.

Gestational age (days)	-1.51 (-12.3, 9.33)	10.7	0.785	Vasodilator Antihypertensive Drugs targeting KCNJ11 (1 SNV)	Wald ratio	rs1557765
Birthweight for gestational age z-score	0.556 (-0.323, 1.44)	0.869	0.215	Vasodilator Antihypertensive Drugs targeting KCNJ11 (1 SNV)	Wald ratio	rs1557765
Birth length (cm)	0.286 (-1.86, 2.43)	2.12	0.794	Vasodilator Antihypertensive Drugs targeting KCNJ11 (1 SNV)	Wald ratio	rs1557765
Head circumference (cm)	-0.0337 (-1.46, 1.39)	1.41	0.963	Vasodilator Antihypertensive Drugs targeting KCNJ11 (1 SNV)	Wald ratio	rs1557765
Apgar score, 1 minute	-0.590 (-1.61, 0.428)	1.01	0.256	Vasodilator Antihypertensive Drugs targeting KCNJ11 (1 SNV)	Wald ratio	rs1557765
Apgar score, 5 minutes	-0.317 (-1.02, 0.390)	0.699	0.380	Vasodilator Antihypertensive Drugs targeting KCNJ11 (1 SNV)	Wald ratio	rs1557765
Hypertensive disorders of pregnancy	3.94 (0.0796, 195)	3.86	0.491	Vasodilator Antihypertensive Drugs targeting KCNJ11 (1 SNV)	Wald ratio	rs1557765
Gestational age (days)	-5.39 (-10.8, 0.0450)	5.37	0.05	Calcium-channel blockers targeting CACNB2 (3 SNVs)	IVW	rs10764331 rs12258967 rs3821843

© 2024 Barry CJS et al. *JAMA Network Open*.

Birthweight for gestational age z-score	0.111 (-0.238, 0.460)	0.345	0.532	Calcium-channel blockers targeting CACNB2 (3 SNVs)	IVW	rs10764331 rs12258967 rs3821843
Birth length (cm)	-0.132 (-1.33, 1.06)	1.18	0.829	Calcium-channel blockers targeting CACNB2 (3 SNVs)	IVW	rs10764331 rs12258967 rs3821843
Head circumference (cm)	-0.0675 (-0.633, 0.498)	0.560	0.815	Calcium-channel blockers targeting CACNB2 (3 SNVs)	IVW	rs10764331 rs12258967 rs3821843
Apgar score, 1 minute	-0.0318 (-0.434, 0.370)	0.398	0.877	Calcium-channel blockers targeting CACNB2 (3 SNVs)	IVW	rs10764331 rs12258967 rs3821843
Apgar score, 5 minutes	0.0514 (-0.227, 0.330)	0.275	0.717	Calcium-channel blockers targeting CACNB2 (3 SNVs)	IVW	rs10764331 rs12258967 rs3821843
Hypertensive disorders of pregnancy	0.358 (0.0273, 4.70)	2.55	0.435	Calcium-channel blockers targeting CACNB2 (3 SNVs)	IVW	rs10764331 rs12258967 rs3821843
Congenital malformation	0.689 (0.126, 3.76)	1.68	0.667	Calcium-channel blockers targeting CACNB2 (3 SNVs)	IVW	rs10764331 rs12258967 rs3821843
Congenital malformation	0.517 (0.00292, 91.6)	5.12	0.803	Potassium-sparing Diuretics and Aldosterone Antagonists targeting SCNN1D (1 SNV)	Wald ratio	rs1262894

© 2024 Barry CJS et al. *JAMA Network Open*.

Congenital malformation	0.392 (0.00443, 34.7)	4.43	0.682	Vasodilator Antihypertensive Drugs targeting EDNRA (1 SNV)	Wald ratio	rs13143677
Congenital malformation	4.66 (0.0601, 361.7)	4.30	0.488	Vasodilator Antihypertensive Drugs targeting KCNJ11 (1 SNV)	Wald ratio	rs1557765
Congenital malformation	15.4 (0.586, 407)	3.24	0.101	Beta-adrenoceptor Blocking drugs targeting ADRB1 (1 SNV)	Wald ratio	rs1801253

© 2024 Barry CJS et al. *JAMA Network Open*.

eTable 7: The individual and mean *F* statistics for the Wald ratio and IVW estimates respectively to demonstrate the instrument strength of the SNV-exposure relationship.

**Genetic drug target**	**F-statistic**	**No. SNVs**
ADRB1 beta-adrenoceptor blocker target	69.23	1
CACNB2 calcium channel blocker target	90.32	3
SCNN1D potassium-sparing diuretics and aldosterone antagonist target	33.06	1
EDRNA vasodilator antihypertensive drug target	40.01	1
KCNJ11 vasodilator antihypertensive drug target	38.39	1

© 2024 Barry CJS et al. *JAMA Network Open*.

eAppendix 1: STROBE-MR guidelines.

STROBE-MR checklist of recommended items to address in reports of Mendelian randomization studies

Item No.	Section	Checklist item	Page No.	Relevant text from manuscript
1	**TITLE and ABSTRACT**	Indicate Mendelian randomization (MR) as the study's design in the title and/or the abstract if that is a main purpose of the study	N/A	Against journal policy
	**INTRODUCTION**			
2	**Background**	Explain the scientific background and rationale for the reported study. What is the exposure? Is a potential causal relationship between exposure and outcome plausible? Justify why MR is a helpful method to address the study question	6-8	Profound physiologic changes occur during pregnancy, such as increased body weight, renal blood flow and cardiac output (6–8). These have been found and theorised to impact the pharmacokinetics of many drugs, affecting the distribution, absorption, and metabolism (7–9). Yet, clinical trials typically exclude pregnant women. Recruiting pregnant women to randomised controlled trials (RCTs) is challenging for ethical and practical reasons. Additionally, experiments using pregnant animals are of limited relevance as there may be species-specific effects, whereby a drug is harmful in some animal species but not in a human or vice-versa (10). As a result, there is relatively little evidence about the effects of drugs in pregnancy, both on women and their offspring. Observational studies on pregnant women have found evidence of possible impaired perinatal development due to drug exposure (teratogenic effects) (11–13). However, the data is limited and often conflicting. Clinical guidance is

© 2024 Barry CJS et al. *JAMA Network Open*.

developed using limited available pharmacological evidence, typically with a tendency towards conservative behaviour. Thus, fear of unintentional fetal harm potentially puts mothers and their offspring at risk through medication avoidance or non-adherence (14).

Drug target MR, which uses genetic variants within a gene to proxy a protein drug target can potentially be used to assess drug safety within pregnancy without exposing the foetus to additional risks (20). Here, we are interested in the intrauterine effects of drugs taken by the mother during pregnancy hence we are also using an intergenerational within-family MR design in a large dataset of genotyped parent-offspring trios. If the MR assumptions hold, the maternal genotype is a proxy for prescription drug exposure in utero.

Genetic variants are randomly transmitted from parents to offspring at conception and thus cannot be affected by external factors after conception. However, exposure-related segregation distortion may exist, meaning that environmental factors or downstream expression of variants may influence the probability of a genetic variant being transmitted from the parent via a successful pregnancy (19,21,22). Without related segregation distortion, this random allocation of ‘genetic exposure’ is analogous to the randomisation of treatment within an RCT. As a result, conditional on parental genotype and in the absence of selection bias, offspring genotypes will be independent of the pre-conception environment.

Here, we may instrument drug exposure using genetic variants within genes that relate to the activity of or

© 2024 Barry CJS et al. *JAMA Network Open*.

				encode the protein target of the drug (20). Intergenerational within-family MR uses genetic variants in one generation (e.g., the mother's genotype) as an instrument for maternal exposure to estimate the effects on the offspring (23, 24). Thus, maternal genetic variants related to the activity or expression of a drug target or biomarker may be used to proxy drug exposure effects on infant outcomes to determine evidence of potential teratogenic or beneficial effects, Figure 1 (20,25). MR has been implemented in the literature to identify opportunities for drug repurposing, drug targets and predicting adverse drug effects (20, 26-31) However, few studies have used MR to estimate the intrauterine effects of drugs.
3	**Objectives**	State specific objectives clearly, including pre-specified causal hypotheses (if any). State that MR is a method that, under specific assumptions, intends to estimate causal effects	7	Mendelian randomization (MR) is an instrumental variables analysis in which genetic variants associated with the exposure of interest are used to assess the causal relationships between an exposure and an outcome (19).
	**METHODS**			
4	**Study design and data sources**	Present key elements of the study design early in the article. Consider including a table listing sources of data for all phases of the study. For each data source contributing to the analysis, describe the following:		

© 2024 Barry CJS et al. *JAMA Network Open*.

	a)	Setting: Describe the study design and the underlying population, if possible. Describe the setting, locations, and relevant dates, including periods of recruitment, exposure, follow-up, and data collection, when available.	9, 10, 12	We derived the SNV-outcome associations using individual-level data from the Norwegian Mother, Father and Child Cohort Study (MoBa), a prospective population-based pregnancy cohort study conducted by the Norwegian Institute of Public Health. Pregnant women were recruited at approximately week 18 of gestation across Norway between 1999-2008 (35, 36).<br><br>Data collection occurred at multiple time points within the pregnancy in the form of self-reported questionnaires that were continued after birth. English translations of the questionnaires are available online (37). The MoBa data in this study uses version 12 of the quality-assured data, released in January 2019. Additional information regarding the child's … Genetic data is available for 44,017 mother-father-child trios, with details on genotyping, imputation and quality control available elsewhere (40).<br><br>Summary-level data from a genome-wide association study (GWAS) of systolic blood pressure (SBP) within UK Biobank was used as the exposure dataset, details in **ENote 4**. The sample contained 436,419 male and female participants of European ancestry.
	b)	Participants: Give the eligibility criteria, and the sources and methods of selection of participants. Report the sample size, and whether any power or sample size calculations were carried out prior to the main analysis	9, 12	The cohort includes 114,761 children, 95,248 mothers and 74,626 fathers.<br><br>The sample contained 436,419 male and female participants of European ancestry. We selected the variants that were common to both MoBa and UK Biobank.

© 2024 Barry CJS et al. *JAMA Network Open*.

	c)	Describe measurement, quality control and selection of genetic variants	23	Genotypic data has been passed through a strict quality control pipeline, and batch effects and principal components may be adjusted for; see **eNote 5** (40).
	d)	For each exposure, outcome, and other relevant variables, describe methods of assessment and diagnostic criteria for diseases	10-12	The specific data of interest to this study were from birth information from the MBRN, questionnaire 6 months after birth and genotyped data from mother, father, and offspring trios.<br>A binary measure for "hypertensive disorders of pregnancy" was derived as a positive control. "Hypertensive disorders of pregnancy" was set to "yes" if there was evidence within the following MBRN variables: hypertension in pregnancy, eclampsia, preeclampsia, early preeclampsia, Haemolysis, Elevated Liver enzymes and Low Platelets syndrome (HELLP) (45).<br>The Ages and Stages Questionnaire items in the 6-month questionnaire was used to calculate an offspring developmental score, details in **eNote 3**. Thus, the outcomes of interest were hypertensive disorders of pregnancy, and perinatal birthweight for gestational age z-score (referred to as birthweight z-score), gestational age (days), birth length (cm), head circumference (cm), Apgar score at 1 minute, Apgar score at 5 minutes, developmental score at 6 months and congenital malformation, eTable 3.
	e)	Provide details of ethics committee approval and participant informed consent, if relevant	9	The establishment of MoBa and initial data collection was based on a license from the Norwegian Data Protection Agency and approval from The Regional Committees for Medical and Health Research Ethics. The Norwegian Health Registry Act currently regulates

© 2024 Barry CJS et al. *JAMA Network Open*.

				the MoBa cohort. The current study was approved by The Regional Committees for Medical and Health Research Ethics (2017/1702).
5	**Assumptions**	Explicitly state the three core IV assumptions for the main analysis (relevance, independence and exclusion restriction) as well assumptions for any additional or sensitivity analysis	7, 12	A genetic variant fulfils the criteria of a valid instrument if it (a) is reliably associated with the exposure of interest (relevance) (b), has no uncontrolled common cause with the outcome relationship (independence) and (c), only associates with the outcome via its effect on the exposure of interest (exclusion restriction).<br>To ensure the independence assumption was met, paternal and offspring genotypes were included in the regression alongside the maternal genotype.
6	**Statistical methods: main analysis**	Describe statistical methods and statistics used		
	a)	Describe how quantitative variables were handled in the analyses (i.e., scale, units, model)	12, 13	To derive the SNV-outcome data, we performed linear or logistic regression for continuous or binary outcomes, respectively. Within each model, we controlled for offspring sex, parental age, batch effects and top 20 principal components.<br>Estimates were obtained per 10mmHg lower SBP as this is comparable to the effect of taking an antihypertensive (48).
	b)	Describe how genetic variants were handled in the analyses and, if applicable, how their weights were selected	10-12	Using the National Health Service (NHS) dictionary of medicines and devices (dm+d) search on OpenPrescribing, hypertension-related Virtual Medicinal Products (VMPs) were determined via British National Formulary (BNF) code, see **eNote 2** (41,42). BNF codes and corresponding drug subclasses determined to be

© 2024 Barry CJS et al. *JAMA Network Open*.

				relevant to hypertension are listed in Table 1… All SNVs within these genomic regions were extracted from the MoBa trio-dataset from each member (mother, father, offspring).<br>We selected the variants that were common to both MoBa and UK Biobank. We then identified the subset of SNVs that were associated with SBP $(p < 5 \times 10^{-8})$, and then clumped with a linkage disequilibrium threshold of r^2<0.01.
		c) Describe the MR estimator (e.g. two-stage least squares, Wald ratio) and related statistics. Detail the included covariates and, in case of two-sample MR, whether the same covariate set was used for adjustment in the two samples	13	We estimated the effects of a 10mmHg lower SBP on each outcome using the Wald estimator for drug classes with a single SNV. For drug subclasses with multiple SNVs, we first checked the mechanism of action of all genes targeted by each SNV for the drug subclass. If the mechanisms of action were identical, we used the inverse variance weighted (IVW) estimator for each outcome. If the mechanisms of action were conflicting, SNVs were subdivided into their mechanistic groupings.
		d) Explain how missing data were addressed	3	This study used individual level SNV-outcome data from 29,849 MoBa trios with complete genetic and phenotypic information, and summary level SNV-exposure data from 436,419 UK Biobank participants.
		e) If applicable, indicate how multiple testing was addressed	N/A	N/A
7	**Assessment of assumptions**	Describe any methods or prior knowledge used to assess the assumptions or justify their validity	13, 19	For drug subclasses with multiple SNVs, we first checked the mechanism of action of all genes targeted by each SNV for the drug subclass. If the mechanisms of action were identical, we used the inverse variance weighted (IVW) estimator for each outcome. If the

				mechanisms of action were conflicting, SNVs were subdivided into their mechanistic groupings.<br><br>To identify the potential pleiotropic pathways, we checked if the instruments are associated with other determinants of perinatal outcomes. Within the GWAS catalog, rs1557765 (vasodilator antihypertensive targeting KCNJ11) and rs1801253 (beta-adrenoceptor blocking drugs targeting ADRB1) were associated with BMI, a known determinant of macrosomia and 'large for gestational age' (53,54). We found little evidence of an association between these genetic drug targets and perinatal birthweight z-scores for either parent; however, a reduction in birthweight z-score was found in the offspring genetic drug targets, eTable 6. All other SNVs have only been found to be associated with blood pressure-related measures, which suggests that pleiotropic effects may not strongly influence our results.
8	**Sensitivity analyses and additional analyses**	Describe any sensitivity analyses or additional analyses performed (e.g. comparison of effect estimates from different approaches, independent replication, bias analytic techniques, validation of instruments, simulations)	14, 25	Additionally, we tested the relevance assumption by calculating the individual and mean F-statistics of the instrument-exposure association. An F-statistic greater than 10 is indicative that the model is unlikely to suffer from substantial weak instrument bias (51).<br><br>We could not perform standard MR sensitivity analyses, such as weighted median and weighted mode, to assess the exclusion restriction criterion as these require a larger number of SNVs for the exposure (72). This is a common limitation of drug target MR studies, however, this should be offset against the biological proximity of the genetic variants, which reduces the likelihood of pleiotropic effects (73). Additionally, we were unable to test for heterogeneity, such as

© 2024 Barry CJS et al. *JAMA Network Open*.

					calculating Cochran's Q statistic, due to the small number of SNVs in our analyses after all exclusions and restrictions were applied (74).
9	**Software and pre-registration**				
		a)	Name statistical software and package(s), including version and settings used	13	We used R (version 4.3.0) to analyse the data via the TwoSampleMR package (46,47).
		b)	State whether the study protocol and details were pre-registered (as well as when and where)	N/A	N/A
	**RESULTS**				
10	**Descriptive data**				
		a)	Report the numbers of individuals at each stage of included studies and reasons for exclusion. Consider use of a flow diagram	14	There were 29,849 complete MoBa trios within our study, with mean maternal age 30.2 years and mean paternal age 32.8 years. Of the offspring, 51.1% were male and 48.9% were female. Summary details of outcome measures are available in eTable 3, exclusion criteria are shown in eFigure 1 and a description of the cohort characteristics are available in the cohort profile (35,36).
		b)	Report summary statistics for phenotypic exposure(s), outcome(s), and other relevant variables (e.g. means, SDs, proportions)	14	Summary details of outcome measures are available in eTable 3, exclusion criteria are shown in eFigure 1 and a description of the cohort characteristics are available in the cohort profile (35,36).
		c)	If the data sources include meta-analyses of previous studies, provide	N/A	N/A

© 2024 Barry CJS et al. *JAMA Network Open*.

			the assessments of heterogeneity across these studies		
		d)	For two-sample MR:<br>i. Provide justification of the similarity of the genetic variant-exposure associations between the exposure and outcome samples<br>ii. Provide information on the number of individuals who overlap between the exposure and outcome studies	27	The genetic instruments were selected from a GWAS of systolic blood pressure conducted in UK Biobank, which includes male and female participants aged 40-69. While instruments selected from this GWAS are likely to reflect the broad SBP phenotype, they may miss female-specific or time-dependent effects due to the inclusion of men and older individuals respectively. Female-specific SNVs could be identified by using a GWAS conducted in female participants only. However, we were unable to find a publicly available GWAS for this purpose (76).
11	**Main results**				
		a)	Report the associations between genetic variant and exposure, and between genetic variant and outcome, preferably on an interpretable scale	12, 29	Summary-level data from a genome-wide association study (GWAS) of systolic blood pressure (SBP) within UK Biobank was used as the exposure dataset, details in **eNote 4**.<br>Information on how to access the MoBaPsychGen post-imputation QC data is available here: https://www.fhi.no/en/more/research-centres/psychgen/access-to-genetic-data-after-quality-control-by-the-mobapsychgen-pipeline-v/.
		b)	Report MR estimates of the relationship between exposure and outcome, and the measures of uncertainty from the MR analysis, on an interpretable scale, such as odds ratio or relative risk per SD difference	16-17	We found little evidence that higher levels of maternal SBP acting through the EDNRA vasodilator antihypertensive drug target affected birthweight, with a 10mmHg decrease in SBP estimated to increase birthweight z-score by 0.711 (95% CI: -0.09, 1.51). A 10mmHg decrease in SBP acting via this drug target

© 2024 Barry CJS et al. *JAMA Network Open*.

				and gene increased birth length by 2.03cm (95% CI: 0.06, 3.99).
		c) If relevant, consider translating estimates of relative risk into absolute risk for a meaningful time period	N/A	N/A
		d) Consider plots to visualize results (e.g. forest plot, scatterplot of associations between genetic variants and outcome versus between genetic variants and exposure)	15	Figure 2a-c, **eTable 4**, displays results for the effects of the maternal SBP acting through antihypertensive drug targets on offspring outcomes.
12	**Assessment of assumptions**			
		a) Report the assessment of the validity of the assumptions	12, 14	Each SNV-outcome association was estimated using linear or logistic regression for continuous or binary measures. To ensure the independence assumption was met, paternal and offspring genotypes were included in the regression alongside the maternal genotype.<br>We selected the variants that were common to both MoBa and UK Biobank. We then identified the subset of SNVs that were associated with SBP, and then clumped with a linkage disequilibrium threshold of r2<0.01.<br>Additionally, we tested the relevance assumption by calculating the individual and mean F-statistics of the instrument-exposure association. An F-statistic greater than 10 is indicative that the model is unlikely to suffer from substantial weak instrument bias (51).

© 2024 Barry CJS et al. *JAMA Network Open*.

		b)	Report any additional statistics (e.g., assessments of heterogeneity across genetic variants, such as I^2, Q statistic or E-value)	14, 25	Additionally, we tested the relevance assumption by calculating the individual and mean F-statistics of the instrument-exposure association. An F-statistic greater than 10 is indicative that the model is unlikely to suffer from substantial weak instrument bias (51).<br>Additionally, we were unable to test for heterogeneity, such as calculating Cochran's Q statistic, due to the small number of SNVs in our analyses after all exclusions and restrictions were applied (74).
13	**Sensitivity analyses and additional analyses**				
		a)	Report any sensitivity analyses to assess the robustness of the main results to violations of the assumptions	25	We could not perform standard MR sensitivity analyses, such as weighted median and weighted mode, to assess the exclusion restriction criterion as these require a larger number of SNVs for the exposure (72). This is a common limitation of drug target MR studies, however, this should be offset against the biological proximity of the genetic variants, which reduces the likelihood of pleiotropic effects (73).
		b)	Report results from other sensitivity analyses or additional analyses	17, 18	The effects of SBP via paternal drug targets for treatments of hypertension on offspring outcomes are displayed in eFigure 3 and **eTable 5**. We found little evidence that the higher paternal SBP acting through the KNCJ11 vasodilator antihypertensive drug target or the ADRB1 beta-adrenoceptor blocking drugs affected early infant outcomes…

© 2024 Barry CJS et al. *JAMA Network Open*.

				F-statistics were calculated for the individual instruments and, where multiple instruments were available, averaged across the drug class **eTable 7**. All F-statistics were greater than 10, and mean F-statistics ranged between 33.06 (SCNN1D potassium-sparing diuretics and aldosterone antagonist target) and 90.32 (CACNB2 calcium channel blocker target).
	c)	Report any assessment of direction of causal relationship (e.g., bidirectional MR)	18-19	Further, our negative control exposure, paternal levels of genetic drug targets, suggested that levels of parental SBP acting through the EDNRA vasodilator antihypertensive target had similar effects in mothers and fathers. This suggests these associations are unlikely to be due to intrauterine effects. There was evidence that some genetically proxied maternal and paternal drugs target differentially affected offspring outcomes.
	d)	When relevant, report and compare with estimates from non-MR analyses	22-23	Two relevant RCTs comparing differing target diastolic blood pressure targets, controlled pharmacologically, on the development of maternal hypertension are The Control of Hypertension in Pregnancy Study (CHIPS), additionally estimating associations of severe maternal hypertension on perinatal outcomes in a cohort of women with nonproteinuric pre-existing or gestational hypertension…
	e)	Consider additional plots to visualize results (e.g., leave-one-out analyses)	N/A	N/A
**DISCUSSION**				

© 2024 Barry CJS et al. *JAMA Network Open*.

14	**Key results**	Summarize key results with reference to study objectives	2	Mendelian randomization analysis of maternal genetic variants for systolic blood pressure, acting through drug targets for treatments of hypertension, provided little evidence for causal relationships between multiple antihypertensive drug subclass targets and differential risk of measured perinatal outcomes. Similar paternal and maternal effect estimates suggest differential indirect genetic effects of these drug targets minimally impact the offspring via intrauterine exposure.
15	**Limitations**	Discuss limitations of the study, taking into account the validity of the IV assumptions, other sources of potential bias, and imprecision. Discuss both direction and magnitude of any potential bias and any efforts to address them	24-27	Specifically, within our study lifetime-exposure to maternal genetic variants relates to the intrauterine period for the offspring, with most study outcomes measured immediately at birth. Yet genetic variants exert small lifetime effects relative to typical drug exposure (i.e., larger over a specific period). Therefore, we anticipate our estimated effect sizes may not directly equate to clinical results and encourage interpretation based on the potential direction of effects, not the magnitude. Additionally, individual genetic variants each explain a small proportion of the variation…<br><br>The genetic instruments were selected from a GWAS of systolic blood pressure conducted in UK Biobank, which includes male and female participants aged 40-69. While instruments selected from this GWAS are likely to reflect the broad SBP phenotype, they may miss female-specific or time-dependent effects due to the inclusion of men and older individuals respectively. Female-specific SNVs could be identified by using a GWAS conducted in female participants only. However, we were unable to find a publicly available GWAS for this purpose (76).

© 2024 Barry CJS et al. *JAMA Network Open*.

				Time-dependent effects are a known issue with MR (77).
16	**Interpretation**			
		a) Meaning: Give a cautious overall interpretation of results in the context of their limitations and in comparison with other studies	18-19, 21-23, 24	We found some evidence that higher levels of maternal SBP acting through drug targets for antihypertensives may improve early infant outcomes. Our results for most outcomes suggest that many genetic drug targets, such as the ADRB1 beta-adrenoreceptor blocking target, are unlikely to have large detrimental effects on the infant outcomes within this study. Further, our negative control exposure, paternal levels of genetic drug targets, suggested that levels of parental SBP acting through the EDNRA vasodilator antihypertensive target had similar effects in mothers and fathers. This suggests these associations are unlikely to be due to intrauterine effects. There was evidence that some genetically proxied maternal and paternal drugs target differentially affected offspring outcomes.<br><br>Few antihypertensives within our positive control analysis demonstrated a reduced risk of maternal hypertensive disorders of pregnancy. However, we had limited statistical power to detect effects on this positive control outcome. Furthermore, these instruments were not selected as antihypertensive drug targets using blood pressure measured in pregnant women, or as instruments for pre-eclampsia, and the mechanisms through which they act may differ.<br><br>A recent paper implemented MR to investigate the safety of beta-adrenoreceptor-blocking drugs and calcium channel blockers in pregnancy (31). These drug

© 2024 Barry CJS et al. *JAMA Network Open*.

subclasses are typically the most prescribed antihypertensive drugs during pregnancy. Yet, there is still little evidence from RCTs to evaluate the risks and benefits of use during pregnancy (60,61). They found evidence to suggest that genetically proxied beta-adrenoreceptor blockers may reduce birth weight…We were able to address a limitation of their study through the inclusion of the perinatal and paternal genotypes. Conditioning on the offspring genotype may induce collider bias. However, the inclusion of the paternal genotype likely mitigates this bias. Further, we could exclude pleiotropic effects via the offspring and father…

Two relevant RCTs comparing differing target diastolic blood pressure targets, controlled pharmacologically, on the development of maternal hypertension are The Control of Hypertension in Pregnancy Study (CHIPS), additionally estimating associations of severe maternal hypertension on perinatal outcomes in a cohort of women with nonproteinuric pre-existing or gestational hypertension, or diastolic blood pressure reading 90-150mmHg in clinic, during weeks 14-36 of gestation (62). … We demonstrate little evidence for an effect of maternal SBP acting through the drug targets on congenital malformation in this study. This is in agreement with the literature; for example, a meta-analysis of RCTs and observational studies found no evidence for an increased odds of major congenital abnormalities when considering first-trimester beta adrenoreceptor-blocking drug use vs no use (66). Subsequent studies, further controlling for maternal confounders, also conclude little evidence of congenital

© 2024 Barry CJS et al. *JAMA Network Open*.

				malformations following maternal use of calcium channel blockers, vasodilators and diuretics (16,67,68).<br><br>Here, we estimated the effects of genetically perturbating specific anti-hypertensive drug targets. We are not able to assess the effects of taking a specific drug, which will represent the combined effects of all the drug's targets, the dose, duration, indication timing, etc. However, genetic evidence about the effects of each drug target can be informative about possible effects of perturbating each target.
	b)	Mechanism: Discuss underlying biological mechanisms that could drive a potential causal relationship between the investigated exposure and the outcome, and whether the gene-environment equivalence assumption is reasonable. Use causal language carefully, clarifying that IV estimates may provide causal effects only under certain assumptions	13, 20, 24, 25	We used the paternal genotype as a negative control to determine whether the SNVs were acting on the offspring via the maternal genotype (50). We estimated the paternal effect on the offspring outcome while controlling for the maternal and offspring genotypes. If the effects of variants on the children's outcomes were due to the intrauterine environment, then we would expect the maternal but not the paternal variants to associate with the outcomes.<br><br>Assortative mating creates associations for a number of heritable phenotypes between maternal and paternal genotypes (55). Typically, this inflates the estimated effect through violation of the exclusion restriction criteria if there is assortment on the exposure of interest. However, mothers and fathers are unlikely to assort on protein levels or blood pressure (which is normally unobserved in younger populations). Thus, assortative mating is unlikely to substantially impact our analyses. Evidence from analysis of siblings suggests that

© 2024 Barry CJS et al. *JAMA Network Open*.

			biological traits (e.g., CRP) are less likely to be biased by population or familial effects (56).<br>Second, MR effect estimates are interpreted as the effect of lifetime exposure (69). It is extremely unlikely that maternal levels of these proteins could have large biological effects outside of pregnancy.<br>We could not perform standard MR sensitivity analyses, such as weighted median and weighted mode, to assess the exclusion restriction criterion as these require a larger number of SNVs for the exposure (72). This is a common limitation of drug target MR studies, however, this should be offset against the biological proximity of the genetic variants, which reduces the likelihood of pleiotropic effects (73).
c)	Clinical relevance: Discuss whether the results have clinical or public policy relevance, and to what extent they inform effect sizes of possible interventions	8, 25, 27	Evidence from MR should be triangulated with findings from other study designs to guide clinical decision-making, develop or repurpose drugs or establish drug safety profiles.<br>Therefore, we anticipate our estimated effect sizes may not directly equate to clinical results and encourage interpretation based on the potential direction of effects, not the magnitude. Additionally, individual genetic variants each explain a small proportion of the variation. Thus, although the instruments were above the F-statistic threshold, we may have had insufficient power to detect clinically meaningful effect sizes… We could not exclude small but clinically meaningful effects.<br>This provides an additional source of evidence, which should be triangulated with other observational study designs and RCTs where appropriate, to enhance the

© 2024 Barry CJS et al. *JAMA Network Open*.

				robustness of findings and inform clinical decision-making
17	**Generalizability**	Discuss the generalizability of the study results (a) to other populations, (b) across other exposure periods/timings, and (c) across other levels of exposure	26-27	Although their characteristics generally reflect the demography of Norway, mothers are older and in better health than the population (35,36,75). This may limit the generalisability of our findings and introduce collider bias. However, selection is unlikely based on genetic variants for protein levels, which are typically unknown to participants. There were 918 women in MoBa who reported antihypertensive use at any point within their pregnancy, of which 228 (0.76%) were in our cohort of trios. This is unlikely to have substantially biased our results relative to an untreated population
	**OTHER INFORMATION**			
18	**Funding**	Describe sources of funding and the role of funders in the present study and, if applicable, sources of funding for the databases and original study or studies on which the present study is based	29	This work was supported by the Medical Research Council (MRC) and the University of Bristol MRC Integrative Epidemiology Unit (MC_UU_00011/1, MC_UU_00011/4). CJB is supported by a Wellcome Trust PhD studentship (218495/Z/19/Z). NMD is supported by the Research Council of Norway (295989). AH was supported by the RCN (#274611, #336085) and SENRHA (#2020022). For the purpose of Open Access, the author has applied a CC BY public copyright licence to any Author Accepted Manuscript version arising from this submission.
19	**Data and data sharing**	Provide the data used to perform all analyses or report where and how the data can be accessed, and reference	29	Information on how to access the MoBaPsychGen post-imputation QC data is available here: https://www.fhi.no/en/more/research-

© 2024 Barry CJS et al. *JAMA Network Open*.

		these sources in the article. Provide the statistical code needed to reproduce the results in the article, or report whether the code is publicly accessible and if so, where		centres/psychgen/access-to-genetic-data-after-quality-control-by-the-mobapsychgen-pipeline-v/. Analysis code is available online https://github.com/Ciarrah/MR_MoBa_mat_antihyp.
20	**Conflicts of Interest**	All authors should declare all potential conflicts of interest	29	No funding body has influenced data collection, analysis or interpretation.

This checklist is copyrighted by the Equator Network under the Creative Commons Attribution 3.0 Unported (CC BY 3.0) license (11,12).

eAppendix 2: Sources used to determine antihypertensives of interest to this study.

The National Health Service (NHS) is the publicly funded national healthcare system in the United Kingdom (UK) (13). The dictionary of medicines and devices (dm+d) is a dictionary of descriptions and codes for all licensed medicines and devices used across the NHS, updated weekly (14). Within the dm+d, items are entered as Virtual Medicinal Products (VMPs) (15). A VMP is comprised of a medication, strength and form which is the typical form for primary care instructions within the UK. A VMP comprises a medication, strength and form which is the typical form for primary care instructions within the UK. A VMP search is more inclusive than an Actual Medicinal Product (AMP) search as some AMP items cannot be represented in a way suitable for prescribing due to the number of active ingredients or they contain international non-proprietary name conventions with respect to the UK prescribing system.

OpenPrescribing is an online platform providing information regarding NHS England prescribing patterns, alongside interactive tools that enable users to search for items within the dm+d, further details available elsewhere (16,17). OpenPrescribing is presented using the British National Formulary (BNF), a pharmaceutical reference book collating information from drug manufacturer literature, medical literature, UK health departments and other regulatory and professional bodies). Items in the BNF are given hierarchal codes, first relating to the relevant BNF chapter, then any subdivision, then further sections containing more detailed drug information such as whether it is generic or how it is presented (18).

© 2024 Barry CJS et al. *JAMA Network Open*.

eAppendix 3: Derivation of the prorated developmental score.

The Ages and Stages Questionnaire (ASQ) items from the MoBa 6-month questionnaire was used to measure infant neurodevelopmental status. The 11 maternal reported items focus on motor (e.g., “Does your child roll over from his/her back onto his/her tummy?") and communication skills (e.g., “When you call your child, does he/she turn towards you one of the first times you say his/her name?”), with the following response options and scores:

- “Yes, often”, scored 10
- “Yes, but seldom”, scored 5
- “No, not yet”, scored 0
- “Don’t know”, scored as missing

We calculated a prorated total developmental score (19).

© 2024 Barry CJS et al. *JAMA Network Open*.

eAppendix 4: UK Biobank.

UK Biobank is a population-based health research resource consisting of approximately 500,000 people, aged between 38 years and 73 years, who were recruited between the years 2006 and 2010 from across the UK (20). Particularly focused on identifying determinants of human diseases in middle-aged and older individuals, participants provided a range of information (such as demographics, health status, lifestyle measures, cognitive testing, personality self-report, and physical and mental health measures) via questionnaires and interviews, anthropometric measures, BP readings and samples of blood, urine and saliva were also taken (data available at www.ukbiobank.ac.uk). A full description of the study design, participants and quality control (QC) methods have been described in detail previously (21). UK Biobank received ethical approval from the Research Ethics Committee (REC reference for UK Biobank is 11/NW/0382).

Similar to MoBa, UK Biobank is large-scale and predominantly of white European ancestry (22,23). here are various population demographic differences between UK Biobank and MoBa; for example, the average age in MoBa is 40.8 versus 56.5 years in UK Biobank (25). Despite these differences, we selected the UK Biobank as the second sample for the two-sample MR estimators we used, which must be non-overlapping to avoid biasing the MR estimate towards the observational estimate (24). Our analysis focuses on the maternal genetic variants associated with drug targets for treatments of hypertension that act via SBP, see eTables 3 and 4. Therefore, genetic liability towards SBP, not actual SBP, is relevant to the study. Thus, we feel the population dissimilarity is unlikely to have a major effect (25). Our exposure of interest is systolic blood pressure, and we use maternal genetic variants associated with drug targets for hypertension treatments, see eTables 3 and 4. Furthermore, there is little evidence of heterogeneity in the effects of these variants on blood pressure across these populations or by sex.

It has been established that UK Biobank is prone to selection bias (26). For this study, participants within the UK Biobank are more likely to be on blood pressure-lowering medication; therefore, SNV-exposure associations may be biased as SBP measures for people using medication will be lower than they would otherwise be (27,28).

SBP was ascertained at the initial assessment centre visit using an automated reading Omron device (field ID 4080). Using the MRC IEU GWAS pipeline the SBP GWAS was established (dataset ID ukb-b-20175) (29). The SBP GWAS population contains both male and female participants. Thus, if the mechanism of action of blood-pressure is sex specific, as established by de Ruiter et al in a recent paper, these effects will be diluted and are less likely to reach genome-level significance, and/or will be of reduced magnitude (30). Although there are limitations with the UK Biobank GWAS in this study we were unable to find a female only, large-scale, publicly available SBP GWAS independent of MoBa.

© 2024 Barry CJS et al. *JAMA Network Open*.

eAppendix 5: Quality control of MoBa data.

33,199 individuals in the NORMENT samples were genotyped using Illumina HumanOmniExpress-24v1.0, Illumina InfiniumOmniExpress-24v1.2 and the Illumina Global Screening Array MD v.1.0 + 50k custom OmniExpress overlap content array. 26,990 individuals were genotyped in the ROTTERDAM sample using the Illumina Global Screening Array MD v.1.0 array. 5,410 were sampled in the TED samples using the Illumina InfiniumOmniExpress-24v1.2, and 32,538 were sampled in the HARVEST sample using the llumina HumanCoreExome12v1.1 and Illumina HumanCoreExome24v1.0. Further details regarding pre-imputation QC, phasing and imputation are available elsewhere .

Post-imputation quality control was as follows. Individuals with either reported verses genetic sex mismatch, a sex-chromosome aneuploidy or that were unable to be linked to phenotypic data were excluded from the sample (n=508). The dataset was also checked for Mendelian errors through PLINK's "—mendel" command, meaning the reported parent was incorrect. A threshold of 1% and 5% for the trio and variant error rate respectively was implemented. This resulted in the removal of a further 129 individuals and 1,293 variants. These SNVs on average were less accurately imputed, with mean INFO=0.88 relative to mean INFO=0.97 for all other SNVs. 3,061 individuals were genotyped twice, and 52 individuals were genotyped 3 times to determine the agreement of SNVs within the pairs of duplicated samples. The 2 samples that were indicated as duplicated by MoBa data had low concordance $\hat{\pi} = 0.8$. An additional 2,474 pairs of samples had $0.74 < \hat{\pi} < 0.98$, however are thought to represent the same individual and not reflect sample contamination. Thus, the heterogeneity is likely a result of the inclusion of the same individuals that were genotyped with different chips that impute to differing standards.
140,767 SNVs were excluded for discordance in more than 5% of the duplicated samples.
Finally, one individual from each pair of duplicated was randomly dropped using a seed .

The sample was restricted to individuals of a "European" ancestry using the first 2 principal components of MoBa data.
The MoBa data were merged with the 1000 Genomes reference panel and the 1000 Genomes principal components were projected onto the MoBa data. The principal component values the MoBa samples were compared to each of the populations included in the reference panel. Samples were excluded if their principal component 1 and 2 values were in the range of the non-European samples in the 1000 Genomes reference panel (n=688) (33).

To determine and exclude based on the degree of relatedness the KING was implemented to estimate kinship coefficients. KING was restricted to an independent set of SNVs with MAF>0.10, window=3000kb and LD R^2>0.9 and age was included as a covariate.
This identified 86,175 pairs of known related individuals and 10,769 unknown related individuals. Parental relationships were subsequently updated with these results and families were reconstructed. KING updated the family ID for 24,022 individuals and parental relationships for 21,361 individuals. Where newly assigned relationships appeared to be errors, e.g., parents less than 15 years older than their children, both parents of the same sex, individuals that identify as monozygotic twins but are linked to different pregnancies, and siblings with different parents or an age gap exceeding 25 years, samples were flagged for exclusion (n=375). Further, parent-offspring pairs

© 2024 Barry CJS et al. *JAMA Network Open*.

in which the mother or father identified by KING differed to the genotyped individual specified in the pedigree were flagged for exclusion, reflecting sample mix-up or samples in which the sampled partner is not the biological father. Within the samples of parents and offspring GCTA was used to select an unrelated subsample, with identity by state < 5% after pruning to a set of independent HAPMAP3 SNVs (n=14,092 offspring and n=24,836 unrelated parents) (33).

The first 20 principal components were calculated independently on a subset of the data for both parent and offspring, restricting to variants pruned to independence using PLINK in HAPMAP3. Two sets of principal components were constructed, in which the first used the MoBa data accounting for structure within the data and the second was generated using the 1000 genomes reference panel (33).

© 2024 Barry CJS et al. *JAMA Network Open*.

eFigure 1: An exclusion flowchart demonstrating the cohort derivation.

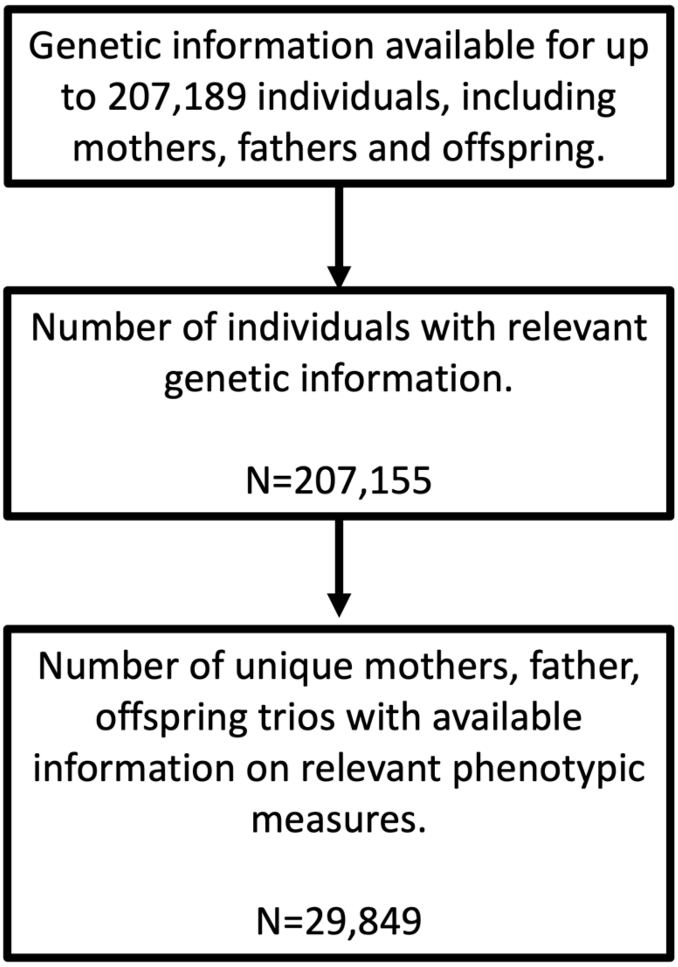

© 2024 Barry CJS et al. *JAMA Network Open*.

eFigure 2: A flowchart describing the instrument derivation procedure.

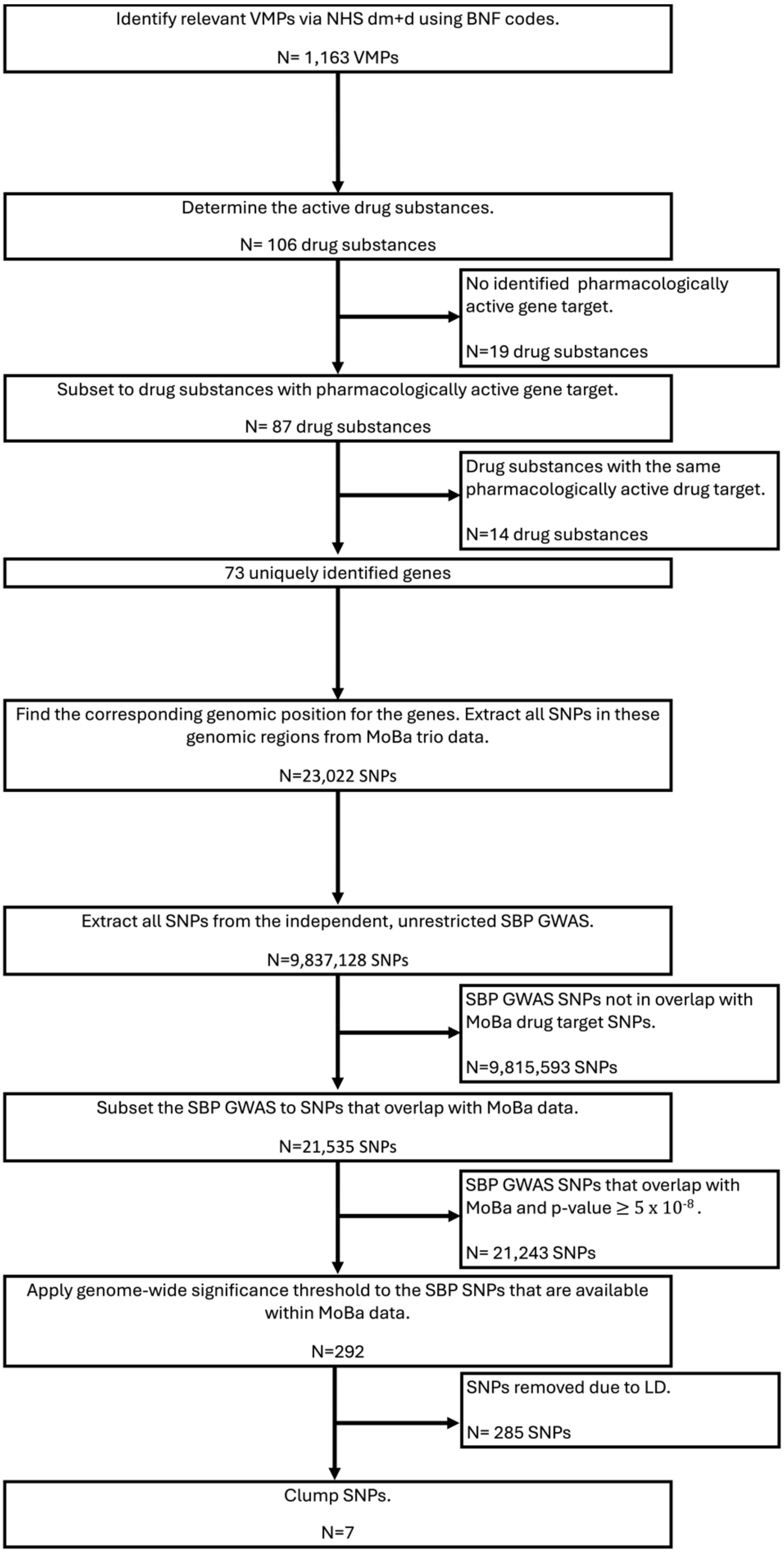

BNF; British National Formulary, MoBa; Norwegian Mother, Father and Child Cohort Study, LD; linkage disequilibrium, NHS; National Health Service, SNV; single-nucleotide variation, VMP; virtual medicine product

© 2024 Barry CJS et al. *JAMA Network Open*.

eFigure 3a, b, c: Forest plots demonstrating the estimated causal effect of the paternal genetic drug targets on offspring outcomes. Results are shown for the IVW estimate where multiple SNVs were available and Wald ratio otherwise. An odds-ratio (OR) has been estimated for the outcomes of “hypertensive disorders of pregnancy” and “congenital malformation”. All other estimates are mean differences.

a)

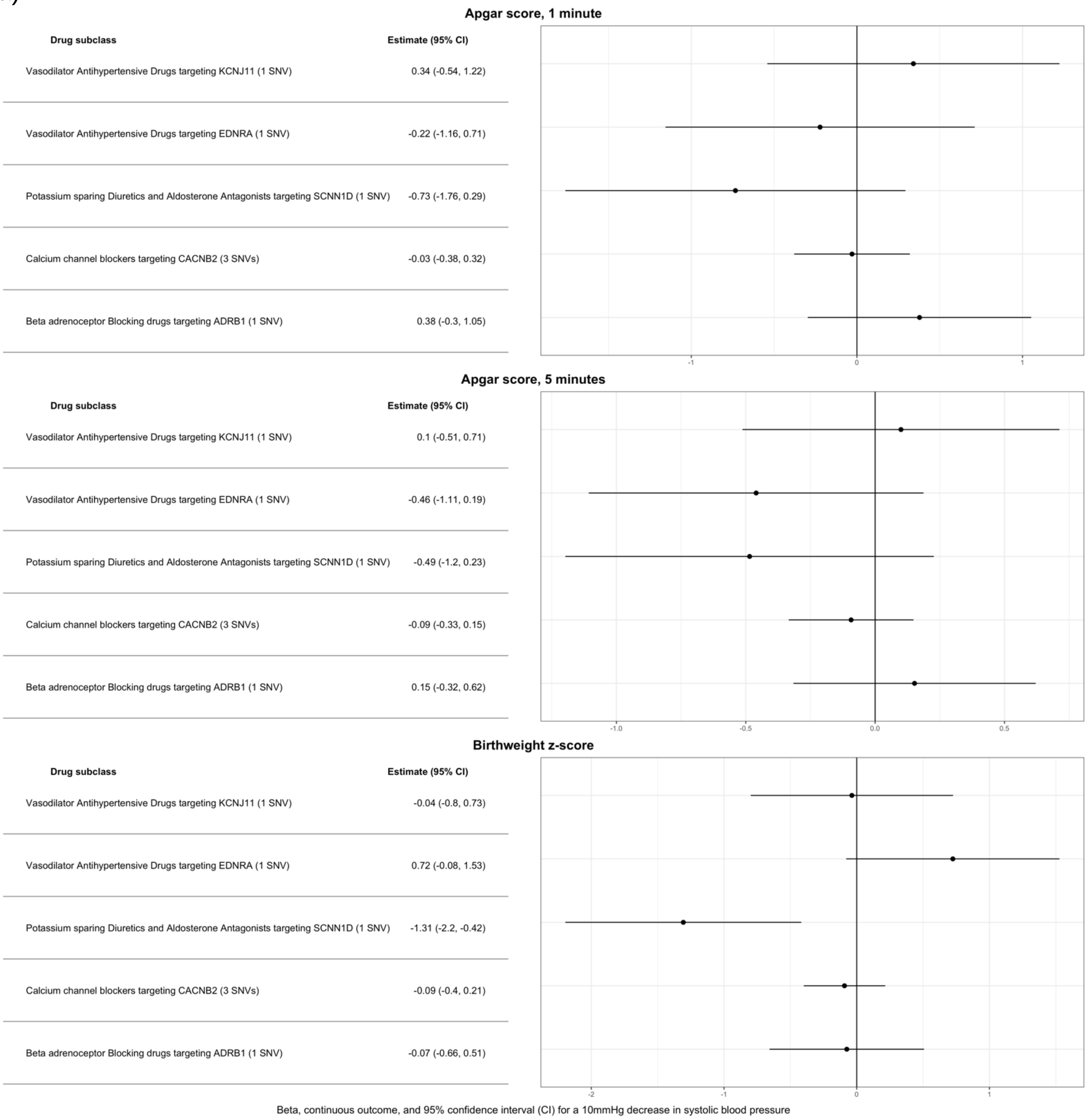

© 2024 Barry CJS et al. *JAMA Network Open*.

b)

Congenital malformation

Drug subclass	Estimate (95% CI)
Vasodilator Antihypertensive Drugs targeting KCNJ11 (1 SNV)	5.77 (0.13, 251.02)
Vasodilator Antihypertensive Drugs targeting EDNRA (1 SNV)	0.42 (0.01, 21.17)
Potassium sparing Diuretics and Aldosterone Antagonists targeting SCNN1D (1 SNV)	0.2 (0, 18.17)
Calcium channel blockers targeting CACNB2 (3 SNVs)	3.58 (0.54, 23.66)
Beta adrenoceptor Blocking drugs targeting ADRB1 (1 SNV)	0.38 (0.02, 6.57)

Developmental score

Drug subclass	Estimate (95% CI)
Vasodilator Antihypertensive Drugs targeting KCNJ11 (1 SNV)	-0.26 (-0.87, 0.36)
Vasodilator Antihypertensive Drugs targeting EDNRA (1 SNV)	-0.59 (-1.24, 0.06)
Potassium sparing Diuretics and Aldosterone Antagonists targeting SCNN1D (1 SNV)	0.15 (-0.57, 0.87)
Calcium channel blockers targeting CACNB2 (3 SNVs)	0.28 (0.03, 0.52)
Beta adrenoceptor Blocking drugs targeting ADRB1 (1 SNV)	-0.01 (-0.48, 0.46)

Gestational age (weeks)

Drug subclass	Estimate (95% CI)
Vasodilator Antihypertensive Drugs targeting KCNJ11 (1 SNV)	-0.78 (-2.15, 0.58)
Vasodilator Antihypertensive Drugs targeting EDNRA (1 SNV)	0.62 (-0.81, 2.06)
Potassium sparing Diuretics and Aldosterone Antagonists targeting SCNN1D (1 SNV)	-0.98 (-2.57, 0.61)
Calcium channel blockers targeting CACNB2 (3 SNVs)	0.46 (-0.44, 1.36)
Beta adrenoceptor Blocking drugs targeting ADRB1 (1 SNV)	-0.23 (-1.27, 0.81)

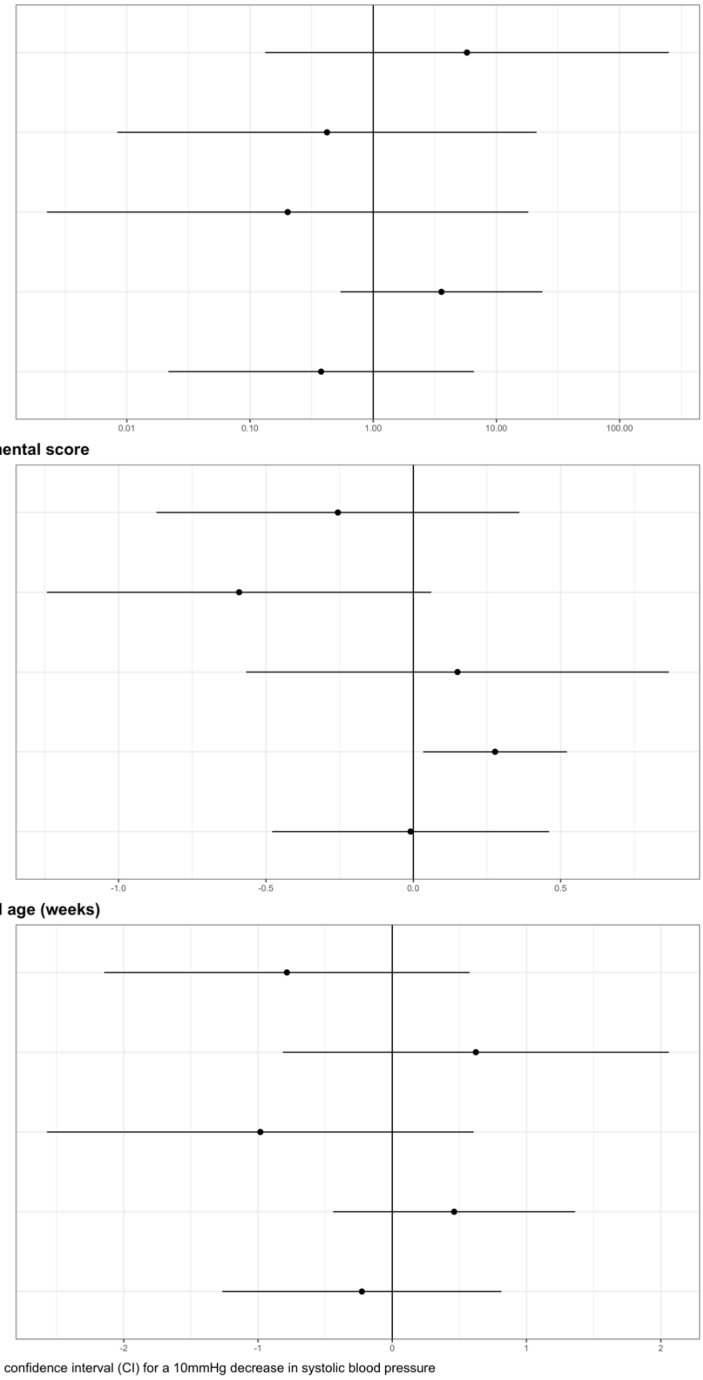

Odds ratio (OR), binary outcome, or beta, continuous outcome, and 95% confidence interval (CI) for a 10mmHg decrease in systolic blood pressure

© 2024 Barry CJS et al. *JAMA Network Open*.

c)

Head circumference (cm)

Drug subclass	Estimate (95% CI)
Vasodilator Antihypertensive Drugs targeting KCNJ11 (1 SNV)	-1.04 (-2.28, 0.19)
Vasodilator Antihypertensive Drugs targeting EDNRA (1 SNV)	0.79 (-0.51, 2.1)
Potassium sparing Diuretics and Aldosterone Antagonists targeting SCNN1D (1 SNV)	-1.63 (-3.07, -0.18)
Calcium channel blockers targeting CACNB2 (3 SNVs)	0.04 (-0.45, 0.53)
Beta adrenoceptor Blocking drugs targeting ADRB1 (1 SNV)	-0.25 (-1.2, 0.69)

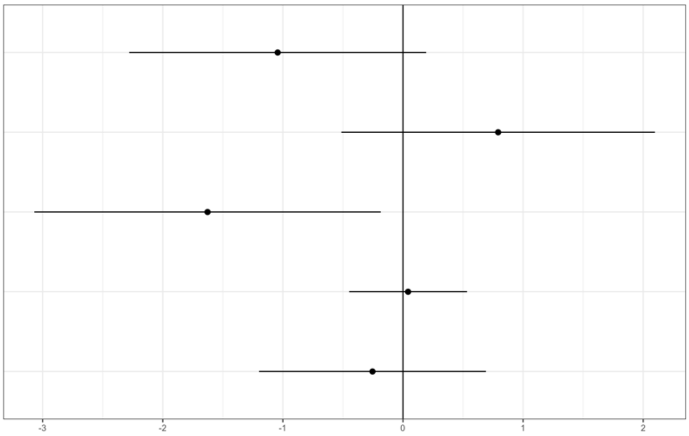

Hypertensive disorders of pregnancy

Drug subclass	Estimate (95% CI)
Vasodilator Antihypertensive Drugs targeting KCNJ11 (1 SNV)	3.13 (0.11, 92.76)
Vasodilator Antihypertensive Drugs targeting EDNRA (1 SNV)	0.87 (0.03, 29.84)
Potassium sparing Diuretics and Aldosterone Antagonists targeting SCNN1D (1 SNV)	3.3 (0.07, 163.59)
Calcium channel blockers targeting CACNB2 (3 SNVs)	1.16 (0.08, 16.4)
Beta adrenoceptor Blocking drugs targeting ADRB1 (1 SNV)	0.29 (0.02, 3.93)

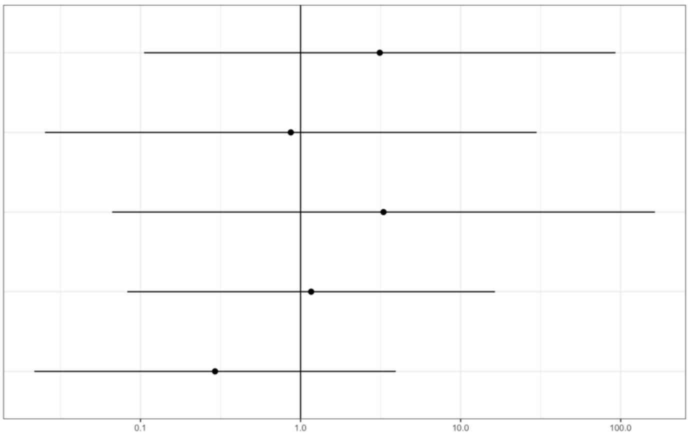

Length at birth (cm)

Drug subclass	Estimate (95% CI)
Vasodilator Antihypertensive Drugs targeting KCNJ11 (1 SNV)	0.4 (-1.46, 2.27)
Vasodilator Antihypertensive Drugs targeting EDNRA (1 SNV)	0.55 (-1.42, 2.51)
Potassium sparing Diuretics and Aldosterone Antagonists targeting SCNN1D (1 SNV)	-1.73 (-3.9, 0.44)
Calcium channel blockers targeting CACNB2 (3 SNVs)	-0.13 (-0.86, 0.61)
Beta adrenoceptor Blocking drugs targeting ADRB1 (1 SNV)	-0.55 (-1.97, 0.87)

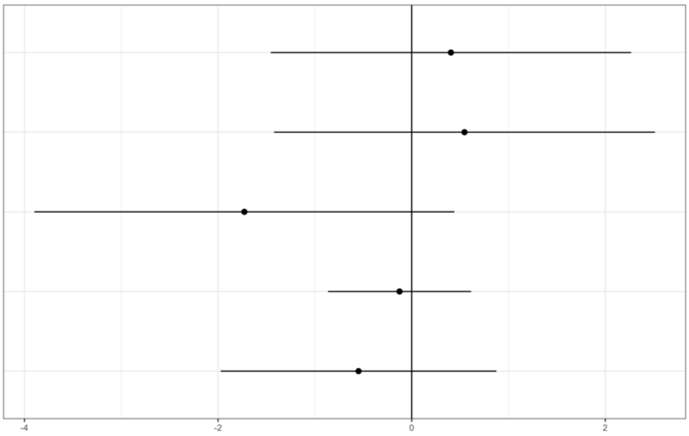

Odds ratio (OR), binary outcome, or beta, continuous outcome, and 95% confidence interval (CI) for a 10mmHg decrease in systolic blood pressure

© 2024 Barry CJS et al. *JAMA Network Open*.

eReferences

1. Duan L, Ng A, Chen W, Spencer HT, Lee MS. Beta-blocker subtypes and risk of low birth weight in newborns. J Clin Hypertens Greenwich Conn. 2018 Nov;20(11):1603–9.

2. Tanaka K, Tanaka H, Kamiya C, Katsuragi S, Sawada M, Tsuritani M, et al. Beta-Blockers and Fetal Growth Restriction in Pregnant Women With Cardiovascular Disease. Circ J Off J Jpn Circ Soc. 2016 Sep 23;80(10):2221–6.

3. Davis RL, Eastman D, McPhillips H, Raebel MA, Andrade SE, Smith D, et al. Risks of congenital malformations and perinatal events among infants exposed to calcium channel and beta-blockers during pregnancy. Pharmacoepidemiol Drug Saf. 2011 Feb;20(2):138–45.

4. Cruickshank DJ, Campbell DM. Atenolol in essential hypertension during pregnancy. BMJ. 1990 Nov 10;301(6760):1103.

5. Bullo M, Tschumi S, Bucher BS, Bianchetti MG, Simonetti GD. Pregnancy outcome following exposure to angiotensin-converting enzyme inhibitors or angiotensin receptor antagonists: a systematic review. Hypertens Dallas Tex 1979. 2012 Aug;60(2):444–50.

6. Moretti ME, Caprara D, Drehuta I, Yeung E, Cheung S, Federico L, et al. The Fetal Safety of Angiotensin Converting Enzyme Inhibitors and Angiotensin II Receptor Blockers. Obstet Gynecol Int. 2012;2012:658310.

7. Cooper WO, Hernandez-Diaz S, Arbogast PG, Dudley JA, Dyer S, Gideon PS, et al. Major congenital malformations after first-trimester exposure to ACE inhibitors. N Engl J Med. 2006 Jun 8;354(23):2443–51.

8. Lennestål R, Otterblad Olausson P, Källén B. Maternal use of antihypertensive drugs in early pregnancy and delivery outcome, notably the presence of congenital heart defects in the infants. Eur J Clin Pharmacol. 2009 Jun;65(6):615–25.

9. Rosenthal T, Oparil S. The effect of antihypertensive drugs on the fetus. J Hum Hypertens. 2002 May;16(5):293–8.

10. Magee LA, Cham C, Waterman EJ, Ohlsson A, von Dadelszen P. Hydralazine for treatment of severe hypertension in pregnancy: meta-analysis. BMJ. 2003 Oct 25;327(7421):955.

11. Skrivankova VW, Richmond RC, Woolf BAR, Davies NM, Swanson SA, VanderWeele TJ, et al. Strengthening the Reporting of Observational Studies in Epidemiology using Mendelian Randomisation (STROBE-MR): Explanation and Elaboration. BMJ. 2021;375:n2233.

12. Skrivankova VW, Richmond RC, Woolf BAR, Yarmolinsky J, Davies NM, Swanson SA, et al. Strengthening the Reporting of Observational Studies in Epidemiology Using Mendelian Randomization: The STROBE-MR Statement. JAMA. 2021 Oct 26;326(16):1614–21.

© 2024 Barry CJS et al. *JAMA Network Open*.

13. Oliver A. The English National Health Service: 1979-2005. Health Econ. 2005;14(S1):S75–99.

14. Dictionary of medicines and devices (dm+d) | NHSBSA [Internet]. [cited 2024 Mar 21]. Available from: https://www.nhsbsa.nhs.uk/pharmacies-gp-practices-and-appliance-contractors/dictionary-medicines-and-devices-dmd

15. Spiers ID. Clinical Terminologies in the NHS: SNOMED CT and dm+d. Br J Pharm [Internet]. 2017 Jul 10 [cited 2024 Mar 21];2(1). Available from: https://www.bjpharm.org.uk/article/id/31/

16. All BNF sections | OpenPrescribing [Internet]. [cited 2022 Mar 9]. Available from: https://openprescribing.net/bnf/

17. About | OpenPrescribing [Internet]. [cited 2022 Sep 23]. Available from: https://openprescribing.net/about/

18. Prescribing Data: BNF Codes | Bennett Institute for Applied Data Science [Internet]. 2017 [cited 2024 Mar 21]. Available from: https://www.bennett.ox.ac.uk/blog/2017/04/prescribing-data-bnf-codes/

19. Graham JW. Missing Data Analysis: Making It Work in the Real World. Annu Rev Psychol. 2009;60(1):549–76.

20. Allen NE, Sudlow C, Peakman T, Collins R, ON BEHALF OF UK BIOBANK. UK Biobank Data: Come and Get It. Sci Transl Med. 2014 Feb 19;6(224):224ed4-224ed4.

21. Collins R. What makes UK Biobank special? The Lancet. 2012 Mar 31;379(9822):1173–4.

22. Sudlow C, Gallacher J, Allen N, Beral V, Burton P, Danesh J, et al. UK Biobank: An Open Access Resource for Identifying the Causes of a Wide Range of Complex Diseases of Middle and Old Age. PLOS Med. 2015 Mar 31;12(3):e1001779.

23. Bycroft C, Freeman C, Petkova D, Band G, Elliott LT, Sharp K, et al. The UK Biobank resource with deep phenotyping and genomic data. Nature. 2018 Oct;562(7726):203–9.

24. Magnus P, Birke C, Vejrup K, Haugan A, Alsaker E, Daltveit AK, et al. Cohort Profile Update: The Norwegian Mother and Child Cohort Study (MoBa). Int J Epidemiol. 2016 Apr;45(2):382–8.

25. Hartwig FP, Davies NM, Hemani G, Davey Smith G. Two-sample Mendelian randomization: avoiding the downsides of a powerful, widely applicable but potentially fallible technique. Int J Epidemiol. 2016 Dec;45(6):1717–26.

26. Schoeler T, Speed D, Porcu E, Pirastu N, Pingault JB, Kutalik Z. Participation bias in the UK Biobank distorts genetic associations and downstream analyses. Nat Hum Behav. 2023 Jul;7(7):1216–27.

© 2024 Barry CJS et al. *JAMA Network Open*.

27. Bias in two-sample Mendelian randomization when using heritable covariable-adjusted summary associations - PMC [Internet]. [cited 2024 Apr 11]. Available from: https://www.ncbi.nlm.nih.gov/pmc/articles/PMC8580279/

28. Wellcome Open Research | Open Access Publishing Platform [Internet]. 2021 [cited 2024 Apr 11]. The consequences of adjustment, correction and selection in ... Available from: https://wellcomeopenresearch.org/articles/6-103/v1

29. Elsworth B, Lyon M, Alexander T, Liu Y, Matthews P, Hallett J, et al. The MRC IEU OpenGWAS data infrastructure [Internet]. bioRxiv; 2020 [cited 2023 Jun 5]. p. 2020.08.10.244293. Available from: https://www.biorxiv.org/content/10.1101/2020.08.10.244293v1

30. de Ruiter SC, Schmidt AF, Grobbee DE, den Ruijter HM, Peters SAE. Sex-specific Mendelian randomisation to assess the causality of sex differences in the effects of risk factors and treatment: spotlight on hypertension. J Hum Hypertens. 2023;37(8):602–8.

31. GitHub [Internet]. [cited 2022 Mar 9]. Projects that have contributed to MoBa Genetics · folkehelseinstituttet/mobagen Wiki. Available from: https://github.com/folkehelseinstituttet/mobagen

32. Helgeland Ø, Vaudel M, Sole-Navais P, Flatley C, Juodakis J, Bacelis J, et al. Characterization of the genetic architecture of BMI in infancy and early childhood reveals age-specific effects and implicates pathways involved in Mendelian obesity [Internet]. medRxiv; 2021 [cited 2022 Mar 9]. p. 2021.05.04.21256508. Available from: https://www.medrxiv.org/content/10.1101/2021.05.04.21256508v1

33. Hughes A, Corfield E, Hemani G, Davies NM, Havdahl A. NIPH PsychGen and MRC IEU post-imputation QC of MoBaGenetics release 1.0, version 1, 27/10/202. :15.

© 2024 Barry CJS et al. *JAMA Network Open*.
