# Supplementary material for: Genetic Insights Into Perinatal Outcomes of Maternal Antihypertensive Therapy During Pregnancy
Source: JAMA Netw Open. 2024 Aug 27;7(8):e2426234. doi: 10.1001/jamanetworkopen.2024.26234 (PMC11350473; doi:10.1001/jamanetworkopen.2024.26234)
Supplement: Supplement 2. — Data Sharing Statement [file jamanetwopen-e2426234-s002.pdf]

Data Sharing Statement

Barry. Genetic Insights Into Perinatal Outcomes of Maternal Antihypertensive Therapy During Pregnancy. *JAMA Netw Open*. Published August 07, 2024.
doi:10.1001/jamanetworkopen.2024.26234

Data
Data available: No

Additional Information
Explanation for why data not available: Individual level data is accessible upon application to MoBa, summary level data is publicly available from IEU OpenGWAS.
